# Time to diagnosis and treatment in younger adults with colorectal cancer: A systematic review

**DOI:** 10.1371/journal.pone.0273396

**Published:** 2022-09-12

**Authors:** Matthew Castelo, Colin Sue-Chue-Lam, Lawrence Paszat, Teruko Kishibe, Adena S. Scheer, Bettina E. Hansen, Nancy N. Baxter

**Affiliations:** 1 Department of Surgery, University of Toronto, Toronto, Ontario, Canada; 2 Institute of Health Policy, Management and Evaluation, Dalla Lana School of Public Health, University of Toronto, Toronto, Ontario, Canada; 3 Li Ka Shing Knowledge Institute, St Michael’s Hospital, Toronto, Ontario, Canada; 4 School of Population and Global Health, University of Melbourne, Melbourne, Victoria, Australia; Ottawa Hospital Research Institute, CANADA

## Abstract

**Background:**

The incidence of colorectal cancer is rising in adults <50 years of age. As a primarily unscreened population, they may have clinically important delays to diagnosis and treatment. This study aimed to review the literature on delay intervals in patients <50 years with colorectal cancer (CRC), and explore associations between longer intervals and outcomes.

**Methods:**

MEDLINE, Embase, and LILACS were searched until December 2, 2021. We included studies published after 1990 reporting any delay interval in adults <50 with CRC. Interval measures and associations with stage at presentation or survival were synthesized and described in a narrative fashion. Risk of bias was assessed using the Newcastle-Ottawa Scale, Institute of Health Economics Case Series Quality Appraisal Checklist, and the Aarhus Checklist for cancer delay studies.

**Results:**

55 studies representing 188,530 younger CRC patients were included. Most studies used primary data collection (64%), and 47% reported a single center. Sixteen unique intervals were measured. The most common interval was symptom onset to diagnosis (21 studies; N = 2,107). By sample size, diagnosis to treatment start was the most reported interval (12 studies; N = 170,463). Four studies examined symptoms onset to treatment start (total interval). The shortest was a mean of 99.5 days and the longest was a median of 217 days. There was substantial heterogeneity in the measurement of intervals, and quality of reporting. Higher-quality studies were more likely to use cancer registries, and be population-based. In four studies reporting the relationship between intervals and cancer stage or survival, there were no clear associations between longer intervals and adverse outcomes.

**Discussion:**

Adults <50 with CRC may have intervals between symptom onset to treatment start greater than 6 months. Studies reporting intervals among younger patients are limited by inconsistent results and heterogeneous reporting. There is insufficient evidence to determine if longer intervals are associated with advanced stage or worse survival.

**Other:**

This study’s protocol was registered with the Prospective Register of Systematic Reviews (PROSPERO; registration number CRD42020179707).

## Introduction

Although the incidence of colorectal cancer (CRC) has been decreasing in older adults, population-based studies in a number of countries have identified that CRC incidence is rising in younger adults (<50 years) [1–3]. Younger patients represent approximately 7% of all new CRC cases [4]. The majority do not have a family history of CRC and only 16% will have an identifiable predisposing factor [4] such as inflammatory bowel disease (IBD) or a polyposis syndrome [5, 6]. Compared to their older counterparts, younger CRC patients present with more advanced disease and more poorly differentiated tumors [4]. Reasons for these disparities are unclear and multifactorial, but delays to diagnosis and treatment have been identified as potential factors [4]. Being a primarily unscreened population, younger adults may experience delays as a consequence of low patient awareness of alarm symptoms, hesitation to seek care, physician misdiagnosis, and poor access to care, resulting in continued tumor growth and advanced stage [4, 7, 8]. The consequences of delay may also differ between younger and older patients with CRC, given differences in tumour biology [4].

**Fig 1:**
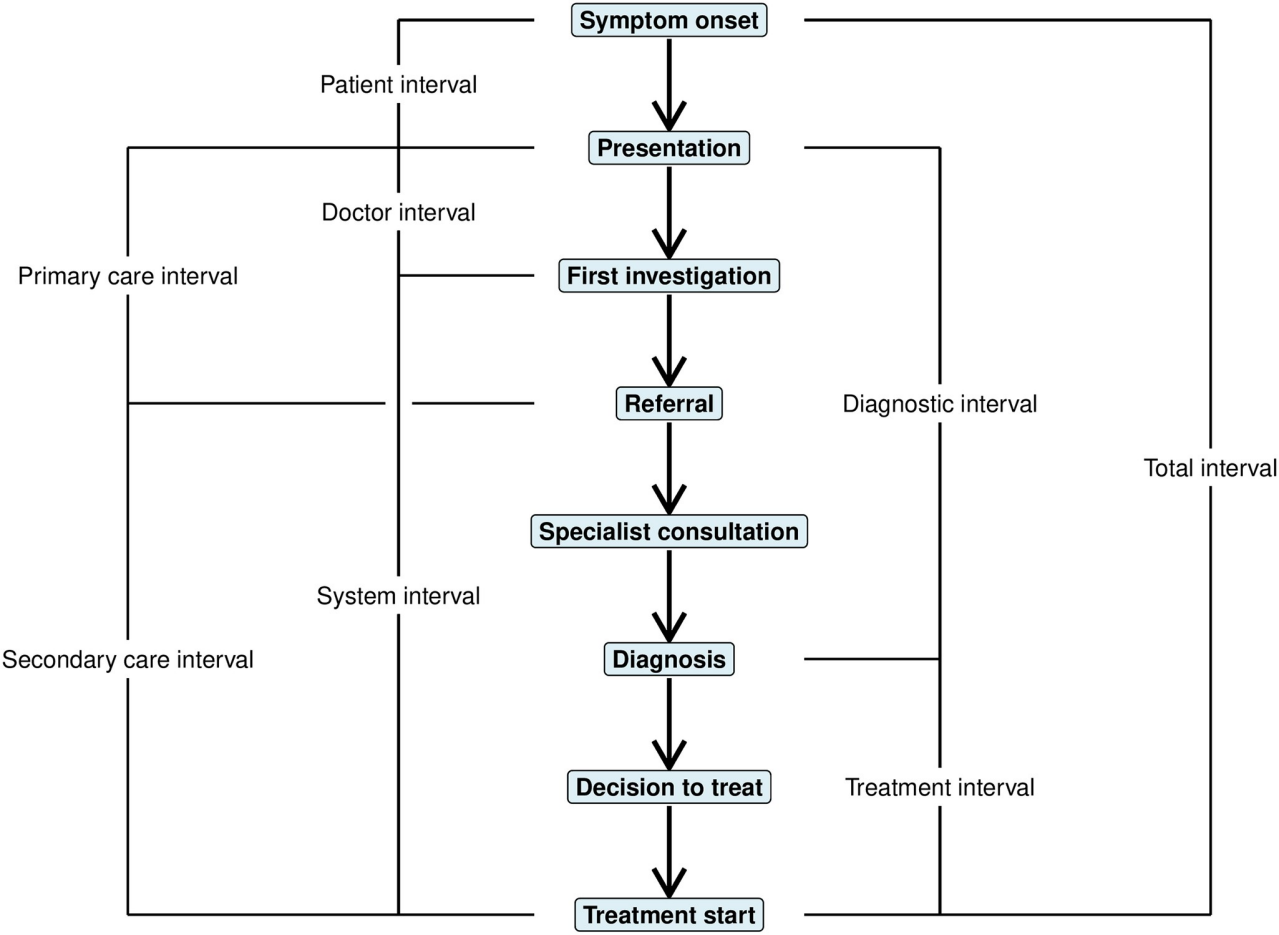
The pathway to treatment. Time points and intervals of interest along the pathway to treatment from symptom onset for patients with colorectal cancer. Intervals are derived from the Aarhus Statement on improving the design and reporting of studies on early cancer diagnosis [10].

Owing to issues of heterogeneous definitions and measurements of delay intervals common across many cancer types, the literature examining the relationship between these intervals and adverse outcomes in CRC cancer is complex to interpret [9]. There has been extensive work to develop a framework to guide research into cancer delays and the measurement of such intervals, culminating in an international consensus document called the Aarhus Statement [10]. The pathway to treatment is conceptualized as a series of milestones beginning with symptom onset, progressing through first contact with the healthcare system, investigation, contact with specialists, and finally cancer diagnosis and treatment initiation [10, 11]. The Aarhus Statement includes standardized definitions of these time points and intervals (Fig 1), and outlines common limitations of study designs. In an effort to improve reporting and conduct among delay studies, the Aarhus consensus group also developed a checklist for evaluating study quality [10].

It is unclear to what extent this checklist, and the Statement more broadly, has penetrated the literature and improved the quality of reporting. Studies focusing on intervals specifically in CRC have mainly considered older adults [9, 12, 13]. In their 2004 review, O’Connell et al. described a number of characteristics of CRC in younger adults, including intervals reported by 42 studies [4]. They concluded mean time to presentation was 6.2 months, although it is unclear how estimates were pooled and no risk of bias assessment was performed [4]. Despite the worsening epidemiologic picture for younger adults with CRC, there has not been an updated attempt to evaluate this literature and describe intervals experienced by younger patients. As poor cancer outcomes in younger patients are often attributed to diagnostic delay [4, 14–19], a systematic review of the literature is needed.

Our aim was to review all observational studies reporting any delay interval among CRC patients <50 years and explore associations between intervals, cancer stage, and survival.

## Methods

We developed a systematic review protocol using the Preferred Reporting Items for Systematic Reviews and Meta-Analyses guidelines for protocols (PRISMA-P) [20]. Our review was prospectively registered on PROSPERO (Prospective Register of Systematic Reviews–registration number CRD42020179707) and is reported according to the PRISMA guidelines [21]. This study used previously published data and did not collect original results, thus patient consent and ethics committee approval were not required.

### Information sources

A search strategy was developed with the assistance of a senior information specialist. The search was limited to observational studies using a published search filter for observational studies [22]. Search results were limited to studies published in four languages (English, French, Portuguese, and Spanish) published from 1990 to the present. This cut-off was chosen to focus on patients receiving more contemporary care. The electronic databases MEDLINE, Embase, and the Latin American and Caribbean Health Sciences Literature (LILACS) were searched from inception until December 2, 2021 (S1 Table). The search strategy was peer reviewed by a second expert information specialist using the Peer Review of Electronic Search Strategies (PRESS) checklist [23]. A grey literature search was performed using the Canadian Agency for Drugs and Technologies in Health Grey Matters checklist (S2 Table) [24].

### Patient and public involvement

Patients were not involved in the design of this study.

### Eligibility criteria

The population of interest was adults <50 years with CRC. We included studies published after 1990 reporting any interval between symptom onset and initiation of treatment among these patients. We included observational studies (retrospective and prospective) in this review. Studies were excluded based on the following criteria: i) intervals not reported stratified by age <50 or younger, ii) majority of patients were pediatric (age <18), iii) only reported intervals including time to adjuvant therapy (i.e. time between surgery and chemotherapy), iv) less than 10 patients <50 years included, v) conference reports, published abstracts without accompanying complete articles, or study protocols, and vi) articles that dealt with delays due to the COVID-19 pandemic.

### Data management

The DistillerSR (Ottawa, Canada) software platform was used to store retrieved articles and perform the study selection process.

### Study selection

Two reviewers (MC and CS) independently screened citations retrieved from the literature search. Screening was conducted in 3 stages: titles, titles and abstracts, and full texts. Conflicts were resolved by discussion, and if required, a third reviewer (NB) was used for adjudication.

### Data collection process

Two reviewers (MC and CS) independently abstracted data. These included study information, patient characteristics, tumor characteristics, interval measures, and cancer outcomes. Conflicts were resolved by discussion and, if required, adjudicated by a third reviewer (NB). Authors were contacted for data clarification as needed.

### Outcomes and definitions

The primary outcomes of interest were interval measures among CRC patients <50. These included the magnitude and variability of any interval falling along the pathway to treatment consistent with the Aarhus Statement, and whether longer intervals were associated with worse survival or advanced stage at presentation. Advanced stage was defined as Stage III or IV, versus Stage I or II.

### Risk of bias assessment

Risk of bias was assessed independently by two reviewers (MC and CS), and conflicts were resolved by discussion. The Newcastle-Ottawa Scale [25] was used to assess the risk of bias for cohort studies that included both younger and older patients with CRC. For studies that examined only younger patients, risk of bias was assessed using the Institute of Health Economics (IHE) Case Series Quality Appraisal Checklist [26].

Further quality appraisal was performed specific to the interval measurement aspect of included studies. We used the Aarhus checklist [10], a 20-item tool designed to evaluate definitions of intervals and their measurement in observational research. The number of applicable items in the Aarhus checklist was determined for each study, and adherence calculated. To explore characteristics associated with higher-quality studies, we contrasted the studies achieving the highest quartile of adherence against the studies in the other quartiles of adherence. These included sample size of younger adults, year of publication, data source, and number of study sites.

### Deviations from the registered protocol

The pre-specified outcome of 5-year survival was modified to include any survival outcome after data extraction was performed. The exploratory analysis concerning adherence to the Aarhus checklist described above was not pre-specified in the systematic review protocol and undertaken *post hoc*.

### Synthesis

Study characteristics and outcomes of interest were described narratively. These included the study definition of younger age, sample size of younger patients, the country of publication, study type, data source, time frame of the study, number of study sites, and language of publication. Each study was categorized according to the intervals measured, by the beginning time point (i.e. symptom onset) and ending time point (i.e. specialist consultation) for the interval. Studies reporting common intervals were grouped and the number of studies and relevant sample sizes of young adults were presented graphically. There was substantial heterogeneity among these interval measures. Therefore, pooling through meta-analysis was not possible. The magnitude of intervals for each study was presented as a lollipop chart, including studies that reported a median or mean length of interval. Time was converted to days for all studies. When studies reported both a median and mean, the median was preferentially plotted and indicated. Studies were grouped by interval and ordered according to decreasing length of interval. Hereditary conditions, predisposing lifestyle factors, tumor biology, and access to care were not consistently reported in a way that enabled inclusion in subgroup analyses. Studies that reported associations between survival or stage at presentation and interval measures for younger patients were described narratively. The number of studies and heterogeneity between them precluded meta-analysis for these outcomes. Data analysis was done in R (R Foundation for Statistical Computing, Vienna, Austria), all statistical tests were two-sided, and *p* < 0.05 was considered statistically significant.

## Results

### Search results

**Fig 2:**
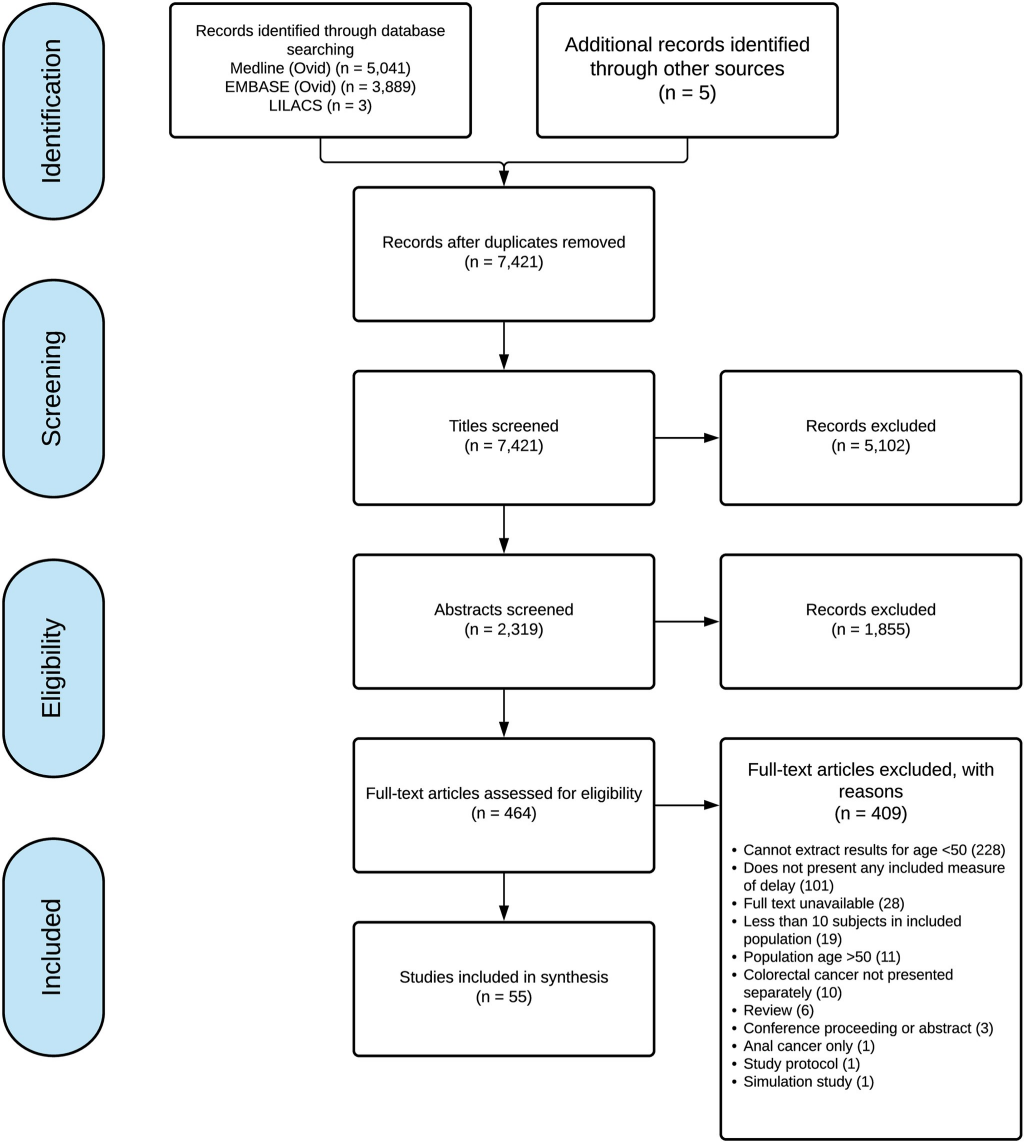
Preferred reporting items for systematic review and meta-analysis flow diagram of included studies.

After duplicates were removed, a total of 7,421 potentially relevant citations were identified from our database and grey literature searches (Fig 2). Full-text evaluation was performed on 464 publications and 55 were included in this review [27–81]. Four were published in French [55, 57, 62, 63], one in Portuguese [81], and the remaining 50 were available in English.

### Study characteristics

Table 1 presents the characteristics of the 55 studies reporting any interval falling along the pathway to treatment (Fig 1) for younger CRC patients. Studies were published between 1992 and 2021, and represent 188,530 young patients (three studies [43, 58, 59] did not report a sample size). The total sample size was heavily skewed by a single very large study published by Gabriel et al. [38] in 2017, which examined colon and rectal cancer patients <50 years compared to patients >60 years using The National Cancer Database. With 96,143 younger colon cancer patients and 58,947 younger rectal cancer patients, this study contributed 82% of all patients in this systematic review [38]. Indeed, of the 52 studies that reported sample sizes, 62% (32/52) had less than 100 younger patients.

**Table 1:** Characteristics of included studies (n = 55) [27–81].

Study	Characteristic							
	Definition of young	N	Country	Study type	Data source	Years of study	Number of sites	Available in
**Colon and rectal cancer**								
Lima 2021 [81]	<50	14,675	Brazil	Retrospective cohort	Primary data collection	2006–2015	Population-based	Portuguese
Johnson 2021 [80]	<50	73	Canada	Retrospective cohort	Primary data collection	2007–2020	1	English
Majano 2021 [79]	<45	131	UK	Retrospective cohort	Cancer registry/health administrative data	2011–2015	Population-based	English
Foppa 2021 [78]	<40	101	Italy	Retrospective cohort	Primary data collection	2008–2019	3	English
Galadima 2021 [71]	<50	522	USA	Retrospective cohort	Cancer registry/health administrative data	2008–2016	Population-based	English
Price 2020 [75]	<50	1206	UK	Retrospective cohort	Cancer registry/health administrative data	2000–2017	Population-based	English
Rittitit 2020 [72]	<50	23	Thailand	Cross-sectional study	Primary data collection	2018	1	English
Delisle 2020 [68]	<50	519	Canada	Retrospective cohort	Cancer registry/health administrative data	2004–2014	Population-based	English
Di Leo 2020 [69]	<50	54	Italy	Retrospective cohort	Primary data collection	2015–2018	1	English
Da Silva 2020 [67]	<50	39	Brazil	Retrospective cohort	Primary data collection	2013–2018	1	English
Webber 2020 [74]	<50	1902	Canada	Retrospective cohort	Cancer registry/health administrative data	2008–2012	Population-based	English
Bergin 2019 [77]	<50	40	Australia	Survey study	Primary data collection and cancer registry	2012–2014	Population-based	English
de Castro 2019 [76]	<50	35	Spain	Retrospective cohort	Primary data collection	2009–2017	1	English
Van Erp 2019 [73]	<50	35	Netherlands	Retrospective cohort	Cancer registry/health administrative data	2007–2011	Population-based	English
Roder 2019 [33]	<50	91	Australia	Retrospective cohort	Cancer registry/health administrative data	2000–2010	4	English
Arhi 2019 [34]	<50	508	UK	Retrospective cohort	Cancer registry/health administrative data	2006–2013	Population-based	English
Kaplan 2019 [35]	20–25	141	Turkey	Retrospective cohort	Primary data collection	2003–2015	20	English
Pearson 2019 [30]	<50	3886	UK	Retrospective cohort	Cancer registry/health administrative data	2014–2015	Population-based	English
Windner 2018 [36]	<50	41	New Zealand	Survey study	Primary data collection	-	-	English
Girolamo 2018 [37]	15–44	3542	UK	Retrospective cohort	Cancer registry/health administrative data	2009–2013	Population-based	English
Rogers 2017 [66]	<50	64	USA	Retrospective cohort	Primary data collection	2008–2010	5	English
Gabriel 2017 [38]	<50	155090	USA	Retrospective cohort	Cancer registry/health administrative data	1998–2011	Population-based	English
Sikdar 2017 [39]	<50	822	Canada	Retrospective cohort	Cancer registry/health administrative data	2004–2010	Population-based	English
Chen 2017 [40]	<50	253	USA	Retrospective cohort	Primary data collection	2008–2014	1	English
Kim 2016 [42]	≤45	693	Republic of Korea	Retrospective cohort	Primary data collection	2006–2011	1	English
Pita-Fernandez 2016 [43]	<50	-	Spain	Retrospective cohort	Primary data collection	1994–2000	1	English
Zhu 2015 [32]	<30	83	China	Retrospective cohort	Primary data collection	1995–2013	1	English
Saluja 2014 [45]	<40	66	India	Retrospective cohort	Primary data collection	2003–2012	1	English
Redaniel 2014 [46]	15–44	921	UK	Retrospective cohort	Cancer registry/health administrative data	1996–2009	Population-based	English
de Sousa 2014 [48]	<50	66	Brazil	Retrospective cohort	Primary data collection	2006–2010	1	English
Esteva 2013 [50]	<50	45	Spain	Cross-sectional study	Primary data collection	2006–2008	5 regions in Spain	English
Taggarshe 2013 [27]	<50	188	USA	Retrospective cohort	Primary data collection	1982–2010	1	English
Kaplan 2013 [51]	20–25	56	Turkey	Retrospective cohort	Primary data collection	2003–2010	9	English
Deng 2012 [52]	<50	75	China	Prospective cohort	Primary data collection	2008–2009	1	English
Mukherji 2011 [53]	<25	32	India	Retrospective cohort	Primary data collection	2000–2006	1	English
Chan 2010 [54]	<40	53	Sri Lanka	Retrospective cohort	Primary data collection	1996–2008	1	English
Fadlouallah 2010 [55]	<40	40	Morocco	Retrospective cohort	Primary data collection	2000–2006	1	French
Shabbir 2009 [56]	<50	38	England	Retrospective cohort	Primary data collection	2001–2005	1	English
Tohme 2008 [57]	<45	43	Lebanon	Retrospective cohort	Primary data collection	1995–2005	1	French
Porter 2005 [58]	<50	-	Canada	Prospective cohort	Primary data collection	2001	1	English
Neal 2005 [59]	<45	-	UK	Survey study	Primary data collection	2002	Population-based	English
Johnston 2004 [60]	25–50	95	Canada	Retrospective cohort	Cancer registry/health administrative data	1992–2000	Population-based	English
Robertson 2004 [61]	<50	53	UK	Retrospective cohort	Cancer registry/health administrative data	1997–1998	Population-based	English
Sahraoui 2000 [62]	<40	88	Morocco	Unclear	Primary data collection	1988–1994	1	French
Pocard 1997 [63]	<40	80	France	Retrospective cohort	Primary data collection	1970–1991	2	French
Heys 1994 [64]	<45	92	UK	Retrospective cohort	Primary data collection	1970–1990	-	English
Marble 1992 [65]	<40	50	USA	Retrospective cohort	Primary data collection	1935–1988	1	English
**Colon cancer**								
Eaglehouse 2020 [70]	<50	664	USA	Retrospective cohort	Cancer registry/health administrative data	1998–2014	Population-based	English
Flemming 2017 [31]	<50	246	Canada	Retrospective cohort	Cancer registry/health administrative data and primary data collection	2002–2008	Population-based	English
Wanis 2017 [29]	<50	47	Canada	Retrospective cohort	Primary data collection	2006–2015	1	English
Jones 2017 [41]	<50	74	USA	Prospective cohort	Primary data collection	2010–2013	9	English
Gillis 2014 [47]	<50	695	Canada	Prospective cohort	Cancer registry/health administrative data	2002–2008	Population-based	English
Ben-Ishay 2013 [49]	<50	31	Israel	Retrospective cohort	Primary data collection	2000–2009	1	English
**Rectal cancer**								
Scott 2016 [28]	<50	56	USA	Case control	Primary data collection	1997–2007	1	English
Zhang 2015 [44]	<50	67	China	Prospective cohort	Primary data collection	2008–2009	1	English

Twenty countries were represented, with the United States (16%, 9/55), United Kingdom (16%, 9/55), and Canada (16%, 9/55) contributing the most studies. Most studies were retrospective cohort studies (78%, 43/55), and used exclusively primary data collection (64%, 35/55). Twenty-five studies (47%) were single center. Six studies (11%) examined colon cancer, two examined rectal cancer (4%), and the remaining 47 (85%) examined both colon and rectal cancer. There was variability in the upper age cut-off for young-onset CRC. Most studies defined the younger cohort as age <50 (67%, 37/55), with the remaining studies using cut-offs from age 45 to age 25 (Table 1).

### Interval measures

**Fig 3:**
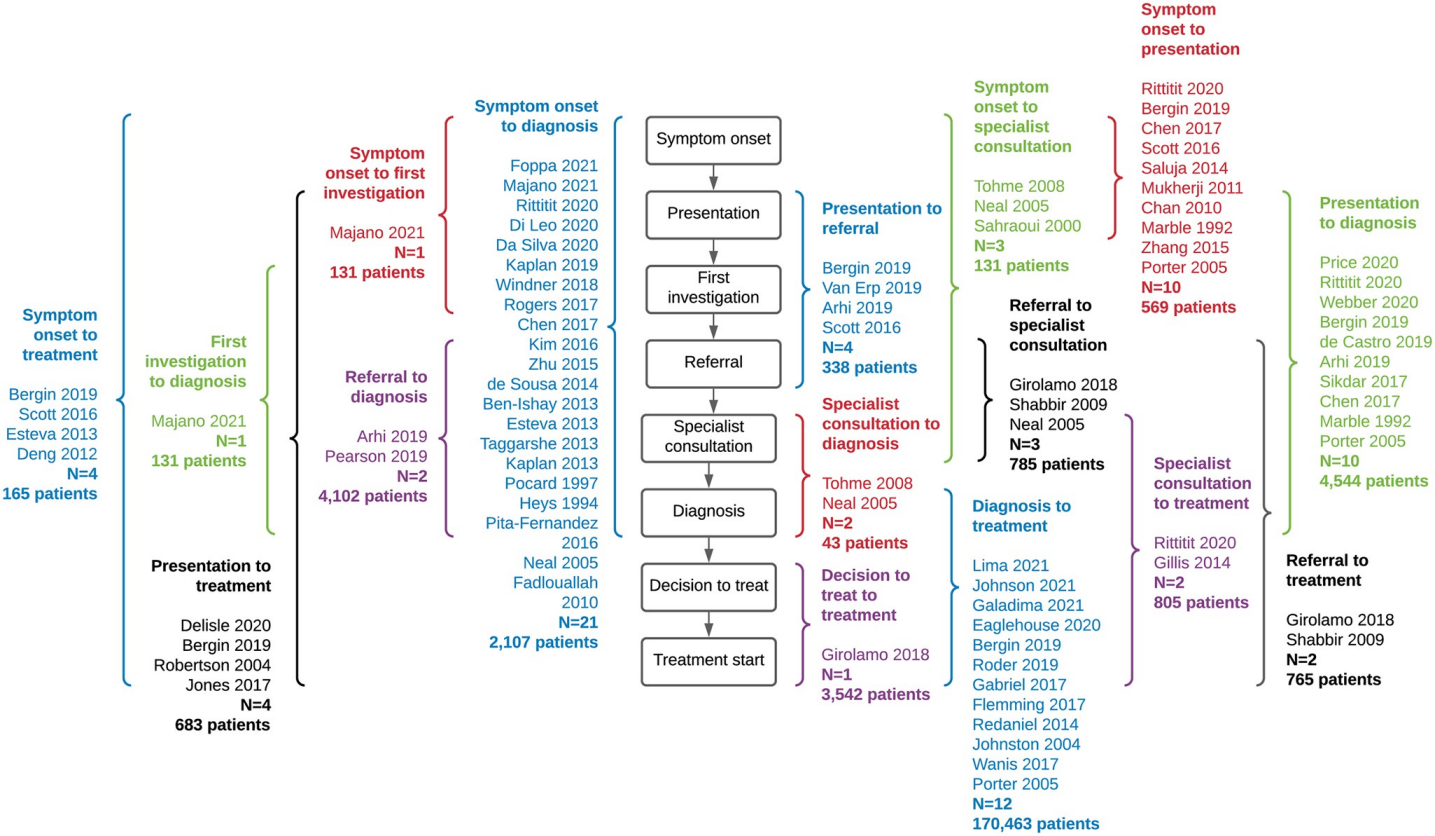
Summary of intervals and sample sizes across studies. Studies are grouped by interval reported, and the total sample size across all studies in each is presented.

Among the 55 included studies, 16 unique intervals were reported (Fig 3) with substantial variation in the number of studies describing each interval. The most common intervals by study number were symptom onset to diagnosis (21 studies), diagnosis to treatment start (12 studies), symptom onset to presentation (10 studies), and presentation to diagnosis (10 studies). Although the largest number of studies examined the interval between symptom onset and diagnosis, these studies included only 2,107 younger patients in total. Due to the presence of the large Gabriel et al. [38] study, which only reported diagnosis to treatment start, this interval was overrepresented with 170,463 younger patients.

**Fig 4:**
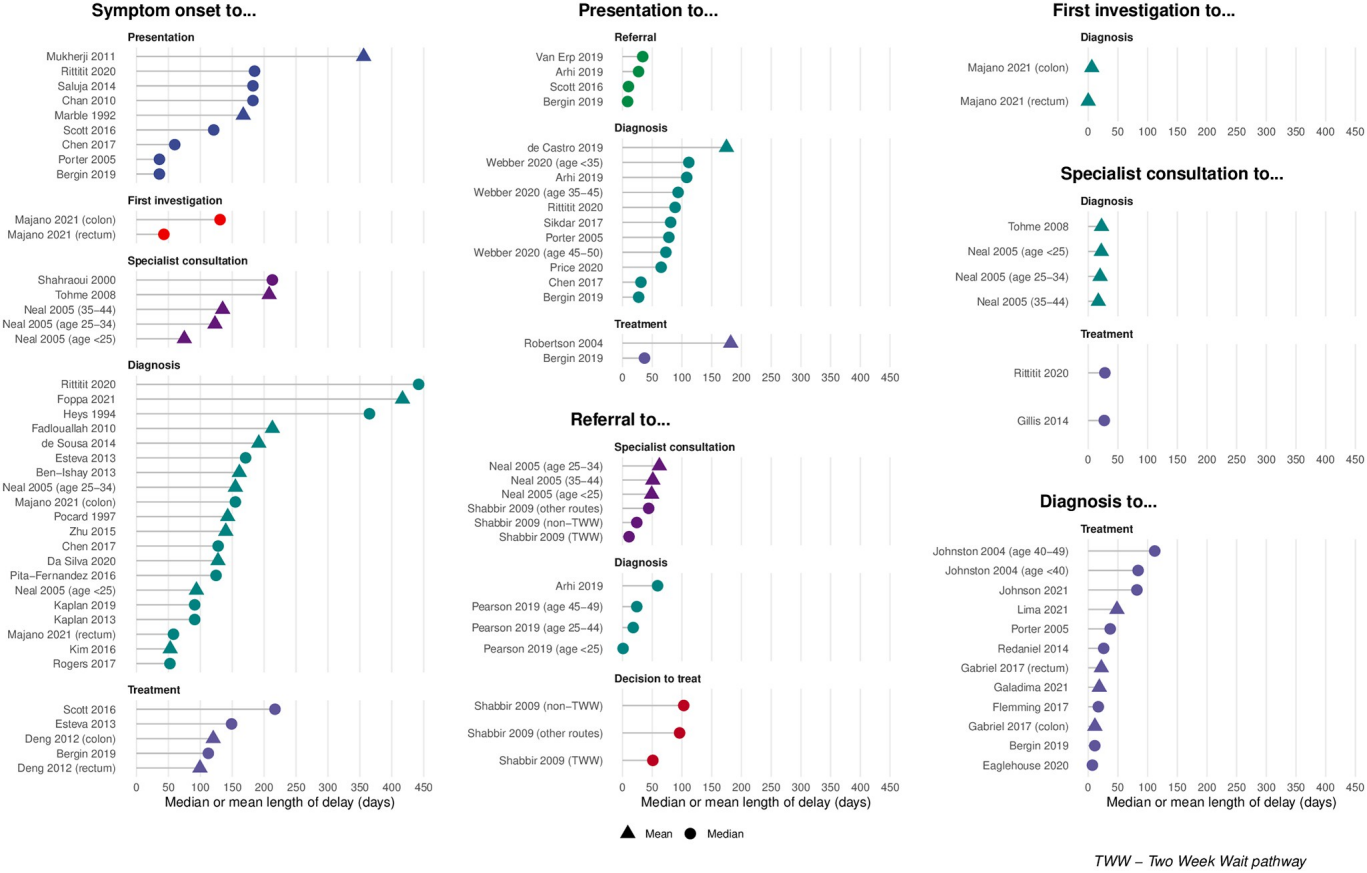
Lengths of unique intervals reported by studies of younger adults with colorectal cancer. Bars represent a single measure from one study, and are color-coded to represent the end of the interval. Circles indicate the median, and triangles the mean. When both were reported by a study, the median was given preference.

There was substantial heterogeneity in reporting of intervals. Raw measures were reported as means, medians, and proportions; many did not include measures of variability (S3 Table). Due to these limitations, pooling of measures was not possible. Individual study measures (means and medians) are presented in Fig 4. There was variability between studies with regards to intervals, particularly those that included time from symptom onset. Studies reported time from symptom onset to presentation ranging from less than 50 days [58, 77] to greater than 350 days [53], and time from symptom onset to diagnosis ranging from approximately 50 days [42, 66] to greater than 400 days [72] (Fig 4). Four studies [28, 50, 52, 77] examined the total interval (symptoms onset to treatment start). The shortest interval was a mean of 99.5 days [52] and the longest was a median of 217 days [28].

Four smaller studies [53, 64, 72, 78] reported intervals greater than 350 days and appeared to be outliers (Fig 4). Mukherji et al. [53] was a single center study of 32 patients <25 years of age in India and reported a mean of 355.9 days from symptoms onset to presentation. Heys et al. [64] reported a median of 365 days from symptoms onset to diagnosis among 92 patients less than 45 years from the UK. Foppa et al. [78] reported mean time from symptoms onset to diagnosis of 416.7 days< for 101 Italian patients <40 years presenting to three tertiary centers. Finally, Rittitit et al. [72] was a single-center study of 23 patients in Thailand and reported the longest interval of any study (median 442 days from symptom onset to diagnosis).

### Risk of bias assessment

The Newcastle-Ottawa scale [25] was used for 47 studies (S4 Table) and the IHE Checklist for Case Series [26] was used for 8 studies (S5 Table). Common sources of bias included low cohort representativeness due to small, single center studies; reporting of loss to follow-up along the pathway to treatment; and not reporting measures of variability for intervals.

The Aarhus Checklist was used to assess risk of bias specific to delay measure studies (S6 Table) [10]. Common limitations of studies included failure to use a theoretical framework to define time points and intervals, poor discussion of bias affecting the measurement of time points (particularly date of symptom onset), not using a hierarchical definition for date of diagnosis, and low precision when describing how time points were ascertained from patient records. Studies reliably defined the beginning and end of intervals and, when relevant, studies reported details of population-based databases (e.g. population coverage).

Based on the Aarhus checklist, the top quartile of adherence consisted of 14 studies (Table 2). All but two [60, 61] were published after the Aarhus Statement. The median percent adherence to the checklist was 86.6% (IQR 83.23–87.5%) in higher-quality studies compared to 20.0% (IQR 11.1–41.2%) in lower-quality studies (*p* <0.001). Higher-quality studies were significantly more likely to use a cancer registry or health administrative database as a data source (85.7% vs. 17.1%, *p* <0.001), and be population-based rather than multi- or single-center (92.9% vs. 17.9%, *p* <0.001).

**Table 2:** Characteristics of higher-quality studies according to the Aarhus checklist.

Study characteristic	Lower quality studies (n = 41) [27–29, 32–36, 38, 40–49, 51–59, 62–67, 69, 71, 72, 76, 78, 80, 81]	Higher quality studies (n = 14) [30, 31, 37, 39, 50, 60, 61, 68, 70, 73–75, 77, 79]	*p*-value
**Percent adherent to checklist (median [IQR])**	20.00 [11.10, 41.20]	86.60 [83.23, 87.50]	<0.001
**Sample size of young colorectal cancer patients (median [IQR])**	70.00 [47.75, 131.00]	382.50 [63.50, 1110.00]	0.054
**Year published, no. (%)**			
**<2012**	11 (26.8)	2 (14.3)	0.556
**2012+**	30 (73.2)	12 (85.7)	
**Data source, no. (%)**			
**Cancer registry/health administrative data**	7 (17.1)	12 (85.7)	<0.001
**Primary data collection**	34 (82.9)	2 (14.3)	
**Number of sites, no. (%)**			
**Population-based**	7 (17.9)	13 (92.9)	<0.001
**Multi-center**	7 (17.9)	1 (7.1)	
**Single-center**	25 (64.1)	0 (0.0)	

Higher-performing defined as highest quartile of percent adherent to applicable Aarhus checklist items.

### Description of higher-quality studies

Flemming et al. [31] examined younger colon cancer patients in Ontario and reported time from diagnosis to treatment (median 17 days). Among CRC patients in Nova Scotia, Johnston et al. [60] reported median time between diagnosis and treatment of 84 days (IQR 63) in those aged <40 and 112 days (IQR 77) in patients aged 40–49. Eaglehouse et al. [70] studied 664 young American CRC patients, reporting only a median 7 days (IQR 18.5) between diagnosis and treatment start.

Webber et al. [74] reported median times from presentation to diagnosis of 111.5 days in patients <35 years, with shorter intervals among patients aged 35–44 and 45–49. Sikdar et al. [39] studied CRC patients in Alberta and reported a median time from presentation to diagnosis of 81 days (n = 822 young patients). Price et al. [75] used linked primary care databases and cancer registries in the UK to measure time from presentation to diagnosis in 1,206 younger CRC patients (median 65 days, IQR 110).

Delisle et al. [68] used population-based data in Manitoba among 519 patients <50 years, with 119 of these classified as having a very long time from presentation to treatment (median 157 days). Robertson et al. [61] included a small number of younger CRC patients (53 patients), showing mean time from presentation to treatment of 182 days.

Pearson et al. [30] was a large (3,886 patients <50) population-based study in the UK that established a methodology for measuring the secondary care diagnostic interval (referral to diagnosis). This interval was a median 1 day (IQR 3) among patients <25 years, 18 days (IQR 53) in patients 25–44 years, and 24 days in patients 45–49 years [30]. Girolamo et al. [37] included 3,542 CRC patients 25–44 years in a multi-cancer UK study and demonstrated the vast majority (98.4%) had a time between the decision to treat and treatment start less than one month, which is a UK waiting time target. Van Erp et al. [73] used linked data between a General Practitioner database and the Netherlands Cancer Registry to estimate time between first presentation and referral (median 34 days) among 35 patients <50 years.

Three higher-quality studies reported an interval containing symptom onset. Esteva et al. [50] contained only 45 younger patients, demonstrating a median 149 days (IQR 110) between symptom onset and treatment. Majano et al. [79] used linked UK databases and was the only study to report the time point of first investigation, showing a median 131 days between symptoms onset to first investigation for younger colon cancer patients, and 43 days for rectal cancer patients. Finally, Bergin et al. [77] reported six intervals resulting from cross-sectional survey data in Australia, including two intervals containing symptom onset. Median time from symptom onset to presentation was 36 days (IQR 76) among 37 younger patients, and time from symptom onset to treatment was a median 113 days (IQR 185) among 34 patients [77].

### Outcomes among younger patients with longer intervals

Few studies reporting intervals among younger patients examined associations between cancer outcomes and length of interval. Four studies [32, 37, 40, 42] reported associations between interval length and advanced stage at presentation, with overall mixed findings (Table 3). They reported populations from four different countries–Korea, UK, USA, and China–representing different healthcare access and delivery models. Kim et al. [42] show significantly increased odds of advanced stage with time from symptoms onset to diagnosis between 1 and 3 months (OR 3.01, 95% CI 1.77–5.12), and greater than 3 months (OR 6.33, 95% CHI 3.05–13.12), compared to less than 1 month. Two studies [32, 37] showed no significant differences in stage at presentation for the intervals between referral and specialist consultation, decision to treat to treatment state, referral to treatment start, and symptom onset to diagnosis. Chen et al. [40] did not report hypothesis tests, but younger patients with late stage at presentation had shorter median intervals between symptoms to presentation, presentation to diagnosis, and symptoms onset to diagnosis.

**Table 3:** Colorectal cancer outcomes (survival and advanced stage at presentation) among younger adults with longer intervals.

Study	Finding	Details
**Kim 2016** [42]	More advanced stage with longer interval	Symptoms to diagnosis, unadjusted
		<1 month: Reference
		1–3 month: OR 3.01 (95% CI 1.77–5.12)
		>3 month: OR 6.33 (95% CI 3.05–13.12)
	Worse survival with longer interval in adjusted analysis only	Symptoms to diagnosis, adjusted cancer-specific survival for sex and tumor differentiation
		<1 month: Reference
		1–3 month: HR 1.62 (95% CI 0.95–2.76)
		>3 month: HR 2.57 (95% CI 1.34–4.94)
		Symptoms to diagnosis, unadjusted cancer-specific survival
		<1 month: Reference
		>3 month: HR 1.69 (95% CI 0.99–2.91)
		1–3 month: HR 1.41 (95% CI 0.86–2.31)
**Girolamo 2018** [37]	No difference or mixed findings for stage	Referral to specialist consultation, unadjusted
		>2 weeks: OR 1.43 (95% CI 0.65–3.52)
		Decision to treat to treatment, unadjusted
		>31 days: OR 0.76 (95% CI 0.43–1.39)
		Referral to treatment, unadjusted
		>62 days: OR 1.03 (95% CI 0.68–1.57)
	No difference or mixed findings for survival	Referral to specialist consultation, unadjusted odds of surviving to one year
		>2 weeks: OR 0.89 (95% CI 0.31–2.57)
		Decision to treat to treatment, unadjusted odds of surviving to one year
		>31 days: OR 0.54 (95% CI 0.17–1.74)
		Referral to treatment, unadjusted odds of surviving to one year
		>62 days: OR 0.50 (95% CI 0.23–1.08)
**Chen 2017** [40]	Less advanced stage with longer interval	Symptom onset to presentation
		Stage I/II: median 90 days
		Stage III/IV: median 60 days
		Presentation to diagnosis
		Stage I/II: median 39 days
		Stage III/IV: median 29 days
		Symptom onset to diagnosis
		Stage I/II: median 129 days
		Stage III/IV: median 89 days
**Zhu 2015** [32]	No difference or mixed findings for stage	Symptom onset to diagnosis
		M0 disease: median 5.6 months
		M1 disease: median 3.0 months, p = 0.101

Two of the four studies also reported the association of survival outcomes with intervals among younger patients (Table 3). In an unadjusted analysis, Kim et al. [42] did not find that younger patients with longer intervals between symptoms onset to diagnosis faced significantly worse survival (interval >3 months HR 1.69, 95% CI 0.99–2.91). Once adjusted for sex and tumor differentiation, an interval greater than 3 months was associated with worse cancer-specific survival (HR 2.57, 95% CI 1.34–4.94). Girolamo et al. [37] did not find any significant associations between survival at one year and three intervals: referral to specialist consultation, referral to treatment start, and decision to treat to treatment start.

## Discussion

This systematic review of 55 observational studies reporting any delay interval among CRC patients <50 years found inconsistent results and substantial heterogeneity with respect to intervals measured, reporting quality, and patient population. Estimates of intervals had high inter-study variability, and there is a paucity of higher-quality literature examining pre-presentation intervals. Younger CRC patients can have time to treatment of 6 months or greater, with much of that contained in the patient interval (symptoms to presentation). It appears once younger adults make contact with the healthcare system, care can be timely, particularly between diagnosis and treatment. Acknowledging the small evidence base and difficulty in studying these associations, there was no clear evidence longer intervals in younger patients were associated with worse survival or more advanced disease at presentation.

There has been long-standing interest in more formally understanding the relationships between intervals, cancer stage at presentation, and ultimately survival [9]. Previous large systematic reviews of mainly older adults have identified a number of methodological challenges with cancer delay studies, and found inconclusive results across a variety of cancers [13]. This review confirms and elaborates on these limitations specific to studies concerning younger adults with CRC. Using the Aarhus Checklist [10], we assessed risk of bias specific to these studies. The most common source of bias was not considering established definitions of intervals and the theoretical frameworks underpinning these definitions. The pathway to treatment is complex–one previous review identified 15 unique intervals [13], while the studies included in this review reported on 16 unique intervals. This heterogeneity precludes formal pooling of interval measures. The included studies also represented twenty countries, with differing patient populations, healthcare access, and healthcare delivery models that may play an important role in determining interval length for younger patients. Further, some jurisdictions have placed particular emphasis on early diagnosis, such as the UK with the Two-Week-Wait referral program. This was observed in the large number of studies originating from the UK (9 studies), with some explicitly aiming to assess the number of patients meeting the two week cut-off from referral to evaluation [37, 56].

We have provided more detail than previous work regarding the relative reporting of different intervals. The patient interval (symptoms onset to presentation) is an important area of study, specifically in younger cancer patients who may not immediately recognize the potential implications of their symptoms and have less routine contact with the health care system [27, 28, 48]. Several included studies reported patient intervals of over 150 days. This interval is greatly underrepresented in the literature compared to primary care and secondary care intervals. This is especially the case among higher-quality studies, which typically take the form of population-based studies utilizing health administrative data. These data sources are generally unable to identify date of symptom onset and are therefore not appropriate for the evaluation of pre-presentation interval. It remains critical to focus on the pre-presentation period when possible.

There is some evidence that younger adults with CRC experience different interval lengths compared to older adults. Our review showed time from diagnosis to treatment can be short among younger adults, and several studies have shown this interval is significantly shorter compared to older patients [31, 38, 46, 81]. Lima et al. [81] reported the odds of the treatment interval being greater than 60 days were significantly higher among those aged 50–59 compared to age <40 (OR 1.32, 95% CI 1.07–1.64; adjusted for race, education, marital status, stage and municipality). Redaniel et al. [46] similarly showed in an adjusted analysis that patients aged 55–64 experienced an additional 2.92 days (95% CI 1.76–4.08) for the treatment interval compared to patients 15–44 years, increasing to an additional 3.76 days (95% CI 2.58–4.93) for those aged 65–74. Studies comparing pre-diagnostic intervals have reached more mixed conclusions. Among 12 studies comparing time between symptom onset and diagnosis between younger and older adults, five [36, 40, 42, 49, 69] found younger adults had significantly longer intervals, two [48, 59] found shorter intervals for younger adults, and the remaining studies showed no significant differences [32, 35, 43, 50, 79]. Dedicated research is needed to further explore these complex comparisons.

Despite the relatively large number of studies included in this review, very few evaluated the impact of longer intervals on cancer outcomes in younger adults. Only two studies [37, 42] explicitly compared survival, and four assessed stage at diagnosis [32, 37, 40, 42], with mixed results. A number of studies and previous reviews hypothesize longer intervals may be contribute to poorer outcomes observed among younger patients [4, 14–19]. We have shown the evidence base specific to younger adults for these assertions is limited, as the existing literature does not show a clear relationship between longer intervals and inferior survival or stage at presentation. In older adults, methodologically robust studies have shown a U-shaped relationship between interval length and mortality, where patients with the shortest and longest intervals have higher mortality [82]. The studies in our review did not explicitly model this relationship, which may have contributed to non-significant findings. While biologically plausible [9], dedicated work is needed to investigate how longer intervals may play important roles in the outcomes of younger CRC patients. This is critical as even nation-wide initiatives such as the Two-Week-Wait referral program in the UK have not reliably translated into improved outcomes for cancer patients, although the potential benefits of early diagnosis cannot be wholly captured in cancer stage and survival [83–88]. Shorter time to diagnosis and treatment may also decrease anxiety and distress among patients and decrease suffering associated with a protracted workup or misdiagnosis.

Strengths of this review include its size, and broad search strategy and inclusion criteria. We included five non-English language studies. This is the largest review reporting intervals among CRC patients younger than 50 years. We used three risk of bias tools, including the Aarhus Checklist [10], developed specifically for delay studies. Using this checklist, we were able to identify and describe high quality studies in this area. Finally, assessment and categorization of intervals was performed in accordance with standardized definitions and frameworks [10].

This work has limitations. We were unable to pool outcomes due to heterogeneity, and this precluded us from making quantitative conclusions regarding the magnitude and impact of interval length among young CRC patients. There was inconsistent reporting of means and medians for intervals, which are often highly skewed, further challenging efforts to compare intervals lengths between studies. However, we identified gaps and common biases in the literature that can provide guidance for future work in this area. There are important patient-level characteristics for younger CRC patients, including hereditary conditions, predisposing lifestyle factors, tumor biology, and access to care that are intrinsically linked to intervals and the diagnostic process. We were unable to incorporate these factors into our review given inter-study variation, differing health contexts, small samples sizes, and sparse reporting.

Given the rising incidence of CRC among younger adults in many jurisdictions and a lack of clear targets for intervention, time to diagnosis and treatment have emerged as potential explanations for disparities in outcomes. This large systematic review has identified a global, broad literature on the subject and concludes that there is still an incomplete understanding of the typical experience of younger CRC patients. The available higher-quality literature is focused mainly on secondary care intervals using population-based data, and the pre-presentation component of intervals remain understudied. Further, the available literature is insufficient to establish whether longer intervals are associated with outcomes in this group.

## Supporting information

S1 Table Search strategy for medline (original search; updated December 2, 2021). (DOCX)

S2 Table Grey Matters checklist tool. (DOCX)

S3 Table Detailed interval measures from individual studies, including measures of variance and sample sizes. (DOCX)

S4 Table Newcastle-Ottawa Scale for cohort studies [25]. Blue indicates full adherence to a scale item, yellow partial adherence, orange minimal adherence, red non-adherence, and gray unclear adherence.
(DOCX)

S5 Table IHE Checklist for Case Series [26]. Blue indicates adherence to a checklist item, orange partial adherence, red non-adherence, and gray unclear adherence.
(DOCX)

S6 Table Aarhus checklist [10]. Red indicates the study did not adhere to the checklist item. Blank cells indicate the checklist item was not applicable to the study.
(DOCX)

S1 Checklist (DOCX)
