# Supplementary material for: Time to diagnosis and treatment in younger adults with colorectal cancer: A systematic review
Source: PLoS One. 2022 Sep 12;17(9):e0273396. doi: 10.1371/journal.pone.0273396 (PMC9467377; doi:10.1371/journal.pone.0273396)
Supplement: S1 Table — (DOCX) [file pone.0273396.s001.docx]

**S1 Table.** Search strategy for Medline (original search; updated December 2, 2021)

## Medline

1 exp Colorectal Neoplasms/ (197733)

2 exp Intestine, Large/ and (Neoplasms/ or Carcinoma/ or Adenocarcinoma/ or Neoplasm Metastasis/) (8771)

3 ((neoplas* or cancer* or carcinom* or adenocarcinoma* or tumo?r* or malignan* or metastas?s) adj7 (colorect* or colon or colons or colonic or colonoscop* or rect* or sigmoid)).tw,kf. (234590)

4 1 or 2 or 3 (280596)

5 Delayed Diagnosis/ (6092)

6 Time-to-Treatment/ (6144)

7 Time Factors/ (1177228)

8 (delay* adj10 (presentation* or referral* or diagnos* or colonoscop* or surg* or treatment* or therap*)).tw. (99597)

9 (delay* adj2 operation*).tw. (958)

10 (patient* adj2 delay*).tw. (9582)

11 (care adj2 delay*).tw. (1975)

12 (system adj2 delay*).tw. (1135)

13 delay*.kf. (10480)

14 (late adj4 (presentation* or diagnos*)).tw,kf. (13985)

15 (postpone* adj3 (presentation* or diagnos* or colonoscop* or surg* or treatment* or therap* or operation*)).tw,kf. (1469)

16 (defer* adj2 (presentation* or diagnos* or colonoscop* or surg* or treatment* or therap* or operation*)).tw,kf. (2490)

17 ((time or timing or timely or untimely) adj2 (presentation* or diagnos* or colonoscop* or surg* or operation* or treatment* or therap*)).tw,kf. (138746)

18 ((interval or intervals) adj4 (presentation* or diagnos* or colonoscop* or surg* or operation* or treatment* or therap*)).tw,kf. (23812)

19 5 or 6 or 7 or 8 or 9 or 10 or 11 or 12 or 13 or 14 or 15 or 16 or 17 or 18 (1428818)

20 Adult/ (4930581)

21 Young Adult/ (818061)

22 Age Factors/ (446214)

23 (young or younger).tw,kf. (644964)

24 "under the age".tw,kf. (14987)

25 "aged under".tw,kf. (3385)

26 early onset.tw,kf. (36991)

27 20 or 21 or 22 or 23 or 24 or 25 or 26 (5639767)

28 comparative study/ (1857196)

29 Follow-Up Studies/ (636777)

30 chang$.tw. (3047736)

31 evaluat$.tw. (3425310)

32 reviewed.tw. (509355)

33 prospective$.tw. (685031)

34 retrospective$.tw. (718864)

35 baseline.tw. (550273)

36 cohort.tw. (518683)

37 consecutive$.tw. (427381)

38 (compare$ or compara$).tw. (4321963)

39 28 or 29 or 30 or 31 or 32 or 33 or 34 or 35 or 36 or 37 or 38 (10743106)

40 4 and 19 and 27 and 39 (5403)

41 limit 40 to yr="1990 -Current" (4963)

42 limit 41 to (english or french or portuguese or spanish) (4566)

## Embase

1 exp colon tumor/ (322524)

2 exp rectum tumor/ (255568)

3 exp Large intestine/ and (neoplasm/ or carcinoma/ or adenocarcinoma/ or metastasis/) (17593)

4 ((neoplas* or cancer* or carcinom* or adenocarcinoma* or tumo?r* or malignan* or metastas?s) adj7 (colorect* or colon or colons or colonic or colonoscop* or rect* or sigmoid)).tw,kw. (345707)

5 1 or 2 or 3 or 4 (441185)

6 therapy delay/ (12498)

7 delayed diagnosis/ (12359)

8 time to treatment/ (16145)

9 (delay* adj10 (presentation* or referral* or diagnos* or colonoscop* or surg* or treatment* or therap*)).tw. (154932)

10 (delay* adj2 operation*).tw. (1438)

11 (patient* adj2 delay*).tw. (15661)

12 (care adj2 delay*).tw. (2994)

13 (system adj2 delay*).tw. (1427)

14 delay*.kw. (25578)

15 (late adj4 (presentation* or diagnos*)).tw,kw. (22468)

16 (postpone* adj3 (presentation* or diagnos* or colonoscop* or surg* or treatment* or therap* or operation*)).tw,kw. (2661)

17 (defer* adj2 (presentation* or diagnos* or colonoscop* or surg* or treatment* or therap* or operation*)).tw,kw. (4029)

18 ((time or timing or timely or untimely) adj2 (presentation* or diagnos* or colonoscop* or surg* or operation* or treatment* or therap*)).tw,kw. (239211)

19 ((interval or intervals) adj4 (presentation* or diagnos* or colonoscop* or surg* or operation* or treatment* or therap*)).tw,kw. (38151)

20 6 or 7 or 8 or 9 or 10 or 11 or 12 or 13 or 14 or 15 or 16 or 17 or 18 or 19 (488406)

21 adult/ (7312139)

22 young adult/ (337850)

23 age/ (529890)

24 (young or younger).tw,kw. (905814)

25 "under the age".tw,kw. (23891)

26 "aged under".tw,kw. (4499)

27 early onset.tw,kw. (54378)

28 21 or 22 or 23 or 24 or 25 or 26 or 27 (8135281)

29 Controlled study/ (7329623)

30 Treatment outcome/ (841244)

31 Major clinical study/ (3740445)

32 (preoperat$ or pre operat$).mp. (536650)

33 chang$.tw. (4124937)

34 evaluat$.tw. (4894802)

35 reviewed.tw. (773638)

36 (compare$ or compara$).tw. (6034025)

37 29 or 30 or 31 or 32 or 33 or 34 or 35 or 36 (17173645)

38 5 and 20 and 28 and 37 (6336)

39 limit 38 to yr="1990 -Current" (6247)

40 limit 39 to (english or french or portuguese or spanish) (6044)

41 limit 40 to embase (3753)

## LILACS

*Database has limited search capabilities; searched using simple and complex combinations of text words
