# Supplementary material for: Time to diagnosis and treatment in younger adults with colorectal cancer: A systematic review
Source: PLoS One. 2022 Sep 12;17(9):e0273396. doi: 10.1371/journal.pone.0273396 (PMC9467377; doi:10.1371/journal.pone.0273396)
Supplement: S3 Table — (DOCX) [file pone.0273396.s003.docx]

**S3 Table.** Detailed interval measures from individual studies, including measures of variance and sample sizes.

| Study | Interval | Mean (days) | Standard deviation | Median (days) | Interquartile range | Proportion | Sample size |
| --- | --- | --- | --- | --- | --- | --- | --- |
| Chen 2017 [40] | Symptom to presentation | 152.0 | 334.2 | 60.0 | 150.0 |  | 253 |
| Scott 2016 [28] | Symptom to presentation |  |  | 121.0 |  |  | 56 |
| Saluja 2014 [45] | Symptom to presentation |  |  | 182.5 |  |  | 66 |
| Mukherji 2011 [53] | Symptom to presentation | 355.9 |  |  |  |  | 32 |
| Chan 2010 [54] | Symptom to presentation | 240.3 |  | 182.5 |  |  | 53 |
| Marble 1992 [65] | Symptom to presentation | 167.3 |  |  |  |  | 50 |
| Zhang 2015 [44] | Symptom to presentation |  |  |  |  | 12 >1 month | 67 |
| Porter 2005 [58] | Symptom to presentation |  |  | 36.0 | 68.0 |  |  |
| Rittitit 2020 [72] | Symptom to presentation | 108.8 | 95% CI 47.6-249.1 | 185 |  |  | 22 |
| Bergin 2019 [77] | Symptom to presentation | 67.5 | 92.5 | 35.9 | 76 |  | 37 |
| Majano [79] colon cancer | Symptom to first investigation |  |  | 131 |  |  | 131 |
| Majano [79] rectal cancer | Symptom to first investigation |  |  | 43 |  |  | 131 |
| Majano [79] colon cancer | First investigation to diagnosis |  |  | 6 |  |  | 131 |
| Majano [79] rectal cancer | First investigation to diagnosis |  |  | 0 |  |  | 131 |
| Arhi 2019 [34] | Presentation to referral |  |  | 27.0 | 100.0 |  | 216 |
| Scott 2016 [28] | Presentation to referral |  |  | 10.0 |  |  | 56 |
| Van Erp 2019 [73] | Presentation to referral |  |  | 34 | 232 |  | 35 |
| Bergin 2019 [77] | Presentation to referral | 41.0 | 73.6 | 8.6 | 32.2 |  | 31 |
| Tohme 2008 [57] | Specialist to diagnosis | 22.4 |  |  |  |  | 43 |
| Neal 2005 [59] age <25 | Specialist to diagnosis | 22.0 | 40.0 |  |  |  |  |
| Neal 2005 [59] age 25-34 | Specialist to diagnosis | 20.0 | 36.0 |  |  |  |  |
| Neal 2005 [59] age 35-44 | Specialist to diagnosis | 17.0 | 27.0 |  |  |  |  |
| Girolamo 2018 [37] | Decision to treat to treatment |  |  |  |  | 3485 <1 month | 3542 |
| Girolamo 2018 [37] | Referral to specialist |  |  |  |  | 721 <14 days | 761 |
| Shabbir 2009 [56] TWW | Referral to specialist |  |  | 11.0 |  |  | 9 |
| Shabbir 2009 [56] non TWW | Referral to specialist |  |  | 24.0 |  |  | 8 |
| Shabbir 2009 [56] other route | Referral to specialist |  |  | 44.0 |  |  | 7 |
| Neal 2005 [59] age <25 | Referral to specialist | 49.0 | 70.0 |  |  |  |  |
| Neal 2005 [59] age 25-34 | Referral to specialist | 62.0 | 71.0 |  |  |  |  |
| Neal 2005 [59] age 35-44 | Referral to specialist | 51.0 | 63.0 |  |  |  |  |
| Roder 2019 [33] | Diagnosis to treatment |  |  |  |  | 60 ≤30 days  20 31-60 days  3 61-90 days  8 ≥90 days | 91 |
| Gabriel 2017 [38] colon cancer | Diagnosis to treatment | 11.2 | 26.7 |  |  |  | 94143 |
| Gabriel 2017 [38] rectal cancer | Diagnosis to treatment | 22.0 | 29.2 |  |  |  | 58947 |
| Flemming 2017 [31] | Diagnosis to treatment |  |  | 17.0 |  |  | 246 |
| Redaniel 2014 [46] | Diagnosis to treatment |  |  | 26.0 | 22.0 |  | 921 |
| Johnston 2004 [60] age <40 | Diagnosis to treatment |  |  | 84.0 | 63.0 |  |  |
| Johnston 2004 [60] age 40-49 | Diagnosis to treatment |  |  | 112.0 | 77.0 |  |  |
| Wanis 2017 [29] | Diagnosis to treatment |  |  |  |  | 22 >30 days | 47 |
| Porter 2005 [58] | Diagnosis to treatment |  |  | 37.0 |  |  |  |
| Galadima 2021 [71] | Diagnosis to treatment | 18.6 | 21.2 |  |  |  | 522 |
| Eaglehouse 2020 [70] | Diagnosis to treatment | 12.1 | 16.2 | 7 | 18.5 |  | 664 |
| Bergin 2019 [77] | Diagnosis to treatment | 14.9 | 15.4 | 11 | 22.6 |  | 39 |
| Johnson 2021 [80] | Diagnosis to treatment | 134.3 | 229 | 82 | 127 |  | 73 |
| Lima 2021 [81] | Diagnosis to treatment | 48.4 |  |  |  |  | 14,675 |
| Tohme 2008 [57] | Symptom to specialist | 207.9 |  |  |  |  | 43 |
| Neal 2005 [59] age <25 | Symptom to specialist | 75.0 | 95.0 |  |  |  |  |
| Neal 2005 [59] age 25-34 | Symptom to specialist | 123.0 | 185.0 |  |  |  |  |
| Neal 2005 [59] age 35-44 | Symptom to specialist | 135.0 | 203.0 |  |  |  |  |
| Sahraoui 2000 [62] | Symptom to specialist |  |  | 212.9 |  |  | 88 |
| Gillis 2014 [47] | Specialist to treatment |  |  | 27.0 |  |  | 695 |
| Rittitit 2020 [72] | Specialist to treatment | 20.7 | 95% CI 10.4-41.4 | 28 |  |  | 20 |
| Girolamo 2018 [37] | Referral to treatment |  |  |  |  | 594 <62 days | 741 |
| Shabbir 2009 [56] TWW | Referral to decision to treat |  |  | 51.0 | 101.0 |  | 9 |
| Shabbir 2009 [56] non TWW | Referral to decision to treat |  |  | 103.0 | 131.0 |  | 8 |
| Shabbir 2009 [56] other route | Referral to decision to treat |  |  | 96.0 | 243.0 |  | 7 |
| Arhi 2019 [34] | Presentation to diagnosis |  |  | 108.0 | 165.0 |  | 216 |
| Sikdar 2017 [39] | Presentation to diagnosis |  |  | 81.0 |  |  | 822 |
| Chen 2017 [40] | Presentation to diagnosis | 91.0 | 232.4 | 31.0 |  |  | 253 |
| Marble 1992 [65] | Presentation to diagnosis |  |  |  |  | mean <1 week | 50 |
| Porter 2005 [58] | Presentation to diagnosis |  |  | 78.0 | 154.0 |  |  |
| Webber 2020 [74] age <35 | Presentation to diagnosis |  |  | 111.5 |  |  | 200 |
| Webber 2020 [74] age 35-44 | Presentation to diagnosis |  |  | 93.5 |  |  | 684 |
| Webber 2020 [74] age 45-49 | Presentation to diagnosis |  |  | 73 |  |  | 1018 |
| Rittitit 2020 [72] | Presentation to diagnosis | 95.9 | 95% CI 50.6-181.7 | 88.5 |  |  | 22 |
| Price 2020 [75] | Presentation to diagnosis | 97.3 | 89.9 | 65 | 110 |  | 1206 |
| de Castro 2019 [76] | Presentation to diagnosis | 174.9 | 208.4 |  |  |  | 35 |
| Bergin 2019 [77] | Presentation to diagnosis | 63.5 | 91.3 | 27.1 | 92.8 |  | 38 |
| Robertson 2004 [61] | Presentation to treatment | 182.0 |  |  |  |  | 53 |
| Jones 2017 [41] | Presentation to treatment |  |  |  |  | 34 >60 days | 74 |
| Delisle 2020 [68] | Presentation to treatment |  |  |  |  | 95 very short (median 5 days)  125 short (median 28 days)  97 moderate (median 56 days)  83 long (median 88 days)  119 very long (median 157 days) | 519 |
| Bergin 2019 [77] | Presentation to treatment | 77.0 | 94.1 | 37 | 96.3 |  | 37 |
| Arhi 2019 [34] | Referral to diagnosis |  |  | 59.0 | 70.0 |  | 216 |
| Pearson 2019 [30] age <25 | Referral to diagnosis |  |  | 1.0 | 3.0 |  | 327 |
| Pearson 2019 [30] age 25-44 | Referral to diagnosis |  |  | 18.0 | 53.0 |  | 2035 |
| Pearson 2019 [30] age 45-49 | Referral to diagnosis |  |  | 24.0 | 47.5 |  | 1524 |
| Scott 2016 [28] | Symptom to treatment |  |  | 217.0 |  |  | 56 |
| Esteva 2013 [50] | Symptom to treatment |  |  | 149.0 | 110.0 |  | 45 |
| Deng 2012 [52] colon cancer | Symptom to treatment | 120.3 |  |  |  |  | 26 |
| Deng 2012 [52] rectal cancer | Symptom to treatment | 99.5 |  |  |  |  | 49 |
| Bergin 2019 [77] | Symptom to treatment | 141.3 | 119.6 | 112.6 | 185.3 |  | 34 |
| Kaplan 2019 [35] | Symptom to diagnosis |  |  | 91.3 |  |  | 141 |
| Windner 2018 [36] | Symptom to diagnosis |  |  |  |  | 13 >6 months | 41 |
| Chen 2017 [40] | Symptom to diagnosis | 243.0 | 465.2 | 128.0 | 205.0 |  | 253 |
| Kim 2016 [42] | Symptom to diagnosis | 52.9 |  |  |  |  | 693 |
| Zhu 2015 [32] | Symptom to diagnosis | 139.9 |  |  |  |  | 83 |
| de Sousa 2014 [48] | Symptom to diagnosis | 191.6 | 121.7 |  |  |  | 66 |
| Ben-Ishay 2013 [49] | Symptom to diagnosis | 161.2 |  |  |  |  | 31 |
| Esteva 2013 [50] | Symptom to diagnosis |  |  | 171.0 | 137.0 |  | 45 |
| Taggarshe 2013 [27] | Symptom to diagnosis |  |  |  |  | 14 >6 months | 79 |
| Kaplan 2013 [51] | Symptom to diagnosis |  |  | 91.3 |  |  | 56 |
| Pocard 1997 [63] | Symptom to diagnosis | 143.0 |  |  |  |  | 75 |
| Heys 1994 [64] | Symptom to diagnosis |  |  | 365.0 |  |  | 92 |
| Pita-Fernandez 2016 [43] | Symptom to diagnosis |  |  | 124.7 | 179.5 |  |  |
| Neal 2005 [59] age <25 | Symptom to diagnosis | 94.0 | 86.0 |  |  |  |  |
| Neal 2005 [59] age 25-34 | Symptom to diagnosis | 155.0 | 207.0 |  |  |  |  |
| Neal 2005 [59] age 35-44 | Symptom to diagnosis |  |  |  |  |  |  |
| Fadlouallah 2010 [55] | Symptom to diagnosis | 212.9 |  |  |  |  | 40 |
| Da Silva 2020 [67] | Symptom to diagnosis | 127.8 | 139.9 |  |  |  | 39 |
| Rogers 2017 [66] | Symptom to diagnosis | 183.4 | 312.4 | 52.5 |  |  | 64 |
| Majano [79] colon cancer | Symptom to diagnosis |  |  | 155 |  |  | 131 |
| Majano [79] rectal cancer | Symptom to diagnosis |  |  | 58 |  |  | 131 |
| Foppa [78] 2021 | Symptom to diagnosis | 416.7 | 298.1 |  |  |  | 101 |
| Di Leo 2020 [69] | Symptom to diagnosis |  |  |  |  | 10 0-1 month  12 2-5 months  18 6-12 months  14 >12 months | 54 |
| Rittitit 2020 [72] | Symptom to diagnosis | 321.4 | 95% CI 180.8-571.3 | 442 |  |  | 23 |

TWW: Two-Week Wait
