# Supplementary material for: Time to diagnosis and treatment in younger adults with colorectal cancer: A systematic review
Source: PLoS One. 2022 Sep 12;17(9):e0273396. doi: 10.1371/journal.pone.0273396 (PMC9467377; doi:10.1371/journal.pone.0273396)
Supplement: S4 Table — Blue indicates full adherence to a scale item, yellow partial adherence, orange minimal adherence, red non-adherence, and gray unclear adherence. (DOCX) [file pone.0273396.s004.docx]

**S4 Table.** Newcastle-Ottawa Scale for Cohort Studies [25]. Blue indicates full adherence to a scale item, yellow partial adherence, orange minimal adherence, red non-adherence, and gray unclear adherence.

| **Study** | **Selection** | | | | **Comparability** | **Outcome** | | |
| --- | --- | --- | --- | --- | --- | --- | --- | --- |
|  | Representativeness of the exposed cohort | Selection of the non-exposed cohort | Ascertainment of exposure | Demonstration that outcome of interest was not present at start of study | Comparability of cohorts on the basis of the design or analysis | Assessment of outcome | Was follow-up long enough for outcomes to occur | Adequacy of follow-up of cohorts |
| **Roder 2019** [33] | Somewhat representative of the average interval in the community | Drawn from the same community as the exposed cohort | Secure record (e.g. surgical records) | Yes | Study controls for additional confounding variables | Record linkage | Yes | No statement |
| **Arhi 2019** [34] | Truly representative of the average interval in the community | Drawn from the same community as the exposed cohort | Secure record (e.g. surgical records) | Yes | Study controls for additional confounding variables | Record linkage | Yes | No statement |
| **Kaplan 2019** [35] | Somewhat representative of the average interval in the community | Drawn from a different source | Secure record (e.g. surgical records) | Yes | Confounding not addressed | Independent blind assessment/medical records | Yes | No statement |
| **Windner 2018** [36] | Selected group of users/patients | Drawn from the same community as the exposed cohort | Written self-report | Yes | Confounding not addressed | Self-report | Yes | No statement |
| **Girolamo 2018** [37] | Truly representative of the average interval in the community | Drawn from the same community as the exposed cohort | Secure record (e.g. surgical records) | Yes | Confounding not addressed | Record linkage | Yes | More than 10% lost and no description of those lost |
| **Gabriel 2017** [38] | Somewhat representative of the average interval in the community | Drawn from the same community as the exposed cohort | Secure record (e.g. surgical records) | Yes | Confounding not addressed | Record linkage | Yes | No statement |
| **Flemming 2017** [31] | Somewhat representative of the average interval in the community | Drawn from the same community as the exposed cohort | Secure record (e.g. surgical records) | Yes | Study controls for additional confounding variables | Independent blind assessment/medical records | Yes | Subjects lost to follow-up unlikely to introduce bias (less than 10%) and description of those lost |
| **Sikdar 2017** [39] | Somewhat representative of the average interval in the community | Drawn from the same community as the exposed cohort | Secure record (e.g. surgical records) | Yes | Study controls for additional confounding variables | Record linkage | Yes | More than 10% lost and no description of those lost |
| **Chen 2017** [40] | Selected group of users/patients | Drawn from the same community as the exposed cohort | Secure record (e.g. surgical records) | Yes | Study controls for additional confounding variables | Independent blind assessment/medical records | Yes | No statement |
| **Kim 2016** [42] | Selected group of users/patients | Drawn from the same community as the exposed cohort | Secure record (e.g. surgical records) | Yes | Confounding not addressed | Independent blind assessment/medical records | Yes | No statement |
| **Scott 2016** [28] | Selected group of users/patients | Drawn from the same community as the exposed cohort | Secure record (e.g. surgical records) | Yes | Study controls for additional confounding variables | Independent blind assessment/medical records | Yes | No statement |
| **Zhu 2015** [32] | Selected group of users/patients | Drawn from the same community as the exposed cohort | Secure record (e.g. surgical records) | Yes | Confounding not addressed | Independent blind assessment/medical records | Yes | No statement |
| **Saluja 2014** [45] | Selected group of users/patients | Drawn from the same community as the exposed cohort | Secure record (e.g. surgical records) | Yes | Confounding not addressed | Independent blind assessment/medical records | Yes | No statement |
| **Redaniel 2014** [46] | Somewhat representative of the average interval in the community | Drawn from the same community as the exposed cohort | Secure record (e.g. surgical records) | Yes | Study controls for additional confounding variables | Record linkage | Yes | No statement |
| **Gillis 2014** [47] | Somewhat representative of the average interval in the community | Drawn from the same community as the exposed cohort | Secure record (e.g. surgical records) | Yes | Study controls for additional confounding variables | Record linkage | No | No statement |
| **de Sousa 2014** [48] | Selected group of users/patients | Drawn from the same community as the exposed cohort | Secure record (e.g. surgical records) | Yes | Confounding not addressed | Independent blind assessment/medical records | Yes | No statement |
| **Ben-Ishay 2013** [49] | Selected group of users/patients | Drawn from the same community as the exposed cohort | Secure record (e.g. surgical records) | Yes | Confounding not addressed | Independent blind assessment/medical records | Yes | No statement |
| **Esteva 2013** [50] | Truly representative of the average interval in the community | Drawn from the same community as the exposed cohort | Secure record (e.g. surgical records) | Yes | Confounding not addressed | Independent blind assessment/medical records | Yes | No statement |
| **Deng 2012** [52] | Selected group of users/patients | Drawn from the same community as the exposed cohort | Structured interview | Yes | Confounding not addressed | Self-report | Yes | No statement |
| **Chan 2010** [54] | Selected group of users/patients | Drawn from the same community as the exposed cohort | Secure record (e.g. surgical records) | Yes | Confounding not addressed | Independent blind assessment/medical records | Yes | No statement |
| **Tohme 2008** [57] | Selected group of users/patients | Drawn from the same community as the exposed cohort | Secure record (e.g. surgical records) | Yes | Confounding not addressed | Independent blind assessment/medical records | Yes | No statement |
| **Johnston 2004** [60] | Somewhat representative of the average interval in the community | Drawn from the same community as the exposed cohort | Secure record (e.g. surgical records) | Yes | Study controls for additional confounding variables | Record linkage | Yes | More than 10% lost and no description of those lost |
| **Robertson 2004** [61] | Somewhat representative of the average interval in the community | Drawn from the same community as the exposed cohort | Secure record (e.g. surgical records) | Yes | Study controls for additional confounding variables | Record linkage | Yes | No statement |
| **Marble 1992** [65] | Selected group of users/patients | Drawn from the same community as the exposed cohort | Secure record (e.g. surgical records) | Yes | Confounding not addressed | Independent blind assessment/medical records | Yes | No statement |
| **Pearson 2019** [30] | Somewhat representative of the average interval in the community | Drawn from the same community as the exposed cohort | Secure record (e.g. surgical records) | Yes | Study controls for additional confounding variables | Record linkage | Yes | More than 10% lost and no description of those lost |
| **Wanis 2017** [29] | Selected group of users/patients | Drawn from the same community as the exposed cohort | Secure record (e.g. surgical records) | Yes | Confounding not addressed | Independent blind assessment/medical records | Yes | No statement |
| **Jones 2017** [41] | Somewhat representative of the average interval in the community | Drawn from the same community as the exposed cohort | Structured interview | Yes | Study controls for additional confounding variables | Self-report | Yes | No statement |
| **Pita-Fernandez 2016** [43] | Selected group of users/patients | Drawn from the same community as the exposed cohort | Secure record (e.g. surgical records) | Yes | Confounding not addressed | Independent blind assessment/medical records | Yes | More than 10% lost and no description of those lost |
| **Zhang 2015** [44] | Selected group of users/patients | Drawn from the same community as the exposed cohort | Secure record (e.g. surgical records) | Yes | Confounding not addressed | Self-report | Yes | Subjects lost to follow-up unlikely to introduce bias (less than 10%) and description of those lost |
| **Porter 2005** [58] | Selected group of users/patients | Drawn from the same community as the exposed cohort | Secure record (e.g. surgical records) | Yes | Study controls for additional confounding variables | Self-report | Yes | More than 10% lost and no description of those lost |
| **Neal 2005** [59] | Truly representative of the average interval in the community | Drawn from the same community as the exposed cohort | Written self-report | Yes | Study controls for additional confounding variables | Self-report | Yes | No statement |
| **Da Silva 2020** [67] | Selected group of users/patients | Drawn from the same community as the exposed cohort | Secure record (e.g. surgical records) | Yes | Confounding not addressed | Independent blind assessment/medical records | Yes | No statement |
| **Rogers 2017** [66] | Selected group of users/patients | Drawn from the same community as the exposed cohort | Structured interview | Yes | Confounding not addressed | Self-report | Yes | No statement |
| **Galadima 2021** [71] | Somewhat representative of the average interval in the community | Drawn from the same community as the exposed cohort | Secure record (e.g. surgical records) | Yes | Confounding not addressed | Independent blind assessment/medical records | Yes | No statement |
| **Delisle 2020** [68] | Truly representative of the average interval in the community | Drawn from the same community as the exposed cohort | Secure record (e.g. surgical records) | Yes | Confounding not addressed | Independent blind assessment/medical records | Yes | No statement |
| **Di Leo 2020** [69] | Selected group of users/patients | Drawn from the same community as the exposed cohort | Secure record (e.g. surgical records) | Yes | Study controls for additional confounding variables | Independent blind assessment/medical records | Yes | No statement |
| **Webber 2020** [74] | Truly representative of the average interval in the community | Drawn from the same community as the exposed cohort | Secure record (e.g. surgical records) | Yes | Confounding not addressed | Record linkage | Yes | Subjects lost to follow-up unlikely to introduce bias (less than 10%) and description of those lost |
| **Van Erp 2019** [73] | Truly representative of the average interval in the community | Drawn from the same community as the exposed cohort | Secure record (e.g. surgical records) | Yes | Confounding not addressed | Independent blind assessment/medical records | Yes | Subjects lost to follow-up unlikely to introduce bias (less than 10%) and description of those lost |
| **Eaglehouse 2020** [70] | Somewhat representative of the average interval in the community | Drawn from the same community as the exposed cohort | Secure record (e.g. surgical records) | Yes | Confounding not addressed | Independent blind assessment/medical records | Yes | Subjects lost to follow-up unlikely to introduce bias (less than 10%) and description of those lost |
| **Rittitit 2020** [72] | Selected group of users/patients | Drawn from the same community as the exposed cohort | Secure record (e.g. surgical records) | Yes | Confounding not addressed | Self-report | Yes | Subjects lost to follow-up unlikely to introduce bias (less than 10%) and description of those lost |
| **Price 2020** [75] | Truly representative of the average interval in the community | Drawn from the same community as the exposed cohort | Secure record (e.g. surgical records) | Yes | Confounding not addressed | Independent blind assessment/medical records | Yes | More than 10% lost and no description of those lost |
| **de Castro 2019** [76] | Selected group of users/patients | Drawn from the same community as the exposed cohort | Secure record (e.g. surgical records) | Yes | Confounding not addressed | Independent blind assessment/medical records | Yes | No statement |
| **Bergin 2019** [77] | Somewhat representative of the average interval in the community | Drawn from the same community as the exposed cohort | Written self-report | Yes | Confounding not addressed | Self-report | Yes | No statement |
| **Majano 2021** [79] | Truly representative of the average interval in the community | Drawn from the same community as the exposed cohort | Secure record (e.g. surgical records) | Yes | Study controls for additional confounding variables | Record linkage | Yes | No statement |
| **Foppa 2021** [78] | Somewhat representative of the average interval in the community | Drawn from the same community as the exposed cohort | Secure record (e.g. surgical records) | Yes | Confounding not addressed | Independent blind assessment/medical records | Yes | No statement |
| **Johnson 2021** [80] | Selected group of users/patients | Drawn from the same community as the exposed cohort | Secure record (e.g. surgical records) | Yes | Confounding not addressed | Independent blind assessment/medical records | Yes | No statement |
| **Lima 2021** [81] | Truly representative of the average interval in the community | Drawn from the same community as the exposed cohort | Secure record (e.g. surgical records) | Yes | Study controls for additional confounding variables | Record linkage | Yes | More than 10% lost and no description of those lost |
