# Supplementary material for: Time to diagnosis and treatment in younger adults with colorectal cancer: A systematic review
Source: PLoS One. 2022 Sep 12;17(9):e0273396. doi: 10.1371/journal.pone.0273396 (PMC9467377; doi:10.1371/journal.pone.0273396)
Supplement: S5 Table — Blue indicates adherence to a checklist item, orange partial adherence, red non-adherence, and gray unclear adherence. (DOCX) [file pone.0273396.s005.docx]

**S5 Table.** IHE Checklist for Case Series [26]. Blue indicates adherence to a checklist item, orange partial adherence, red non-adherence, and gray unclear adherence.

| **IHE Checklist for Case Series** | Mukherji 2011 [53] | Taggarshe 2013 [27] | Kaplan 2013 [51] | Shabbir 2009 [56] | Pocard 1997 [63] | Heys 1994 [64] | Fadlouallah 2010 [55] | Sahraoui 2000 [62] |
| --- | --- | --- | --- | --- | --- | --- | --- | --- |
| **Study Objective** |  |  |  |  |  |  |  |  |
| Was the hypothesis/aim/objective of the study clearly stated? | Partial | Yes | Partial | Partial | Yes | Yes | Partial | No |
| **Study Design** |  |  |  |  |  |  |  |  |
| Was the study conducted prospectively? | No | No | No | No | No | No | No | No |
| Were the cases collected in more than one centre? | No | No | Yes | No | Yes | Unclear | No | No |
| Were patients recruited consecutively? | Unclear | Unclear | Unclear | Unclear | Unclear | Unclear | Unclear | Unclear |
| **Study Population** |  |  |  |  |  |  |  |  |
| Were the characteristics of the patients included in the study described? | Yes | Yes | Yes | Partial | Yes | Yes | Yes | Yes |
| Were the eligibility criteria (i.e. inclusion and exclusion criteria) for entry into the study clearly stated? | Partial | Yes | No | No | Yes | No | No | No |
| Did patients enter the study at a similar point in the disease? | No | No | No | No | No | No | No | No |
| **Outcome measure** |  |  |  |  |  |  |  |  |
| Were relevant outcome measures established a priori? | Partial | Partial | Partial | Yes | Partial | Partial | Partial | No |
| Were the relevant outcomes measured using appropriate objective/subjective methods? | Partial | Partial | Yes | Yes | Partial | Partial | Partial | Partial |
| **Statistical Analysis** |  |  |  |  |  |  |  |  |
| Were the statistical tests used to assess the relevant outcomes appropriate? | Unclear | No | Yes | Unclear | Unclear | Unclear | Unclear | Unclear |
| **Results and Conclusions** |  |  |  |  |  |  |  |  |
| Was follow-up long enough for important events and outcomes to occur? | Yes | Yes | Yes | Yes | Yes | Yes | Yes | Yes |
| Were losses to follow-up reported? | No | No | No | No | No | No | No | No |
| Did the study provide estimates of random variability in the data analysis of relevant outcomes? | No | No | Partial | Partial | Partial | Partial | Partial | Partial |
| Were the conclusions of the study supported by the results? | Unclear | Unclear | Unclear | Unclear | Unclear | Unclear | Unclear | Unclear |
| **Competing Interests and Sources of Support** |  |  |  |  |  |  |  |  |
| Were both competing interests and sources of support for the study reported? | No | Partial | Partial | No | No | No | No | No |

Checklist items pertaining to interventions and adverse events related to interventions were omitted as they were not applicable to any of the included studies.
