# Supplementary material for: Time to diagnosis and treatment in younger adults with colorectal cancer: A systematic review
Source: PLoS One. 2022 Sep 12;17(9):e0273396. doi: 10.1371/journal.pone.0273396 (PMC9467377; doi:10.1371/journal.pone.0273396)
Supplement: S6 Table — Red indicates the study did not adhere to the checklist item. Blank cells indicate the checklist item was not applicable to the study. (DOCX) [file pone.0273396.s006.docx]

**S6 Table.** Aarhus checklist [10]. Red indicates the study did not adhere to the checklist item. Blank cells indicate the checklist item was not applicable to the study.

| **Aarhus Checklist** | Roder 2019[33] | Arhi 2019[34] | Kaplan 2019[35] | | | Windner 2018[36] | | Girolamo 2018[37] | | | Gabriel 2017[38] | | | Flemming 2017[31] | | | | | Sikdar 2017[39] | | | | Chen 2017[40] | | | Kim 2016[42] | | | | Scott 2016[28] | | | | Zhu 2015[32] | | Saluja 2014[45] | |  |
| --- | --- | --- | --- | --- | --- | --- | --- | --- | --- | --- | --- | --- | --- | --- | --- | --- | --- | --- | --- | --- | --- | --- | --- | --- | --- | --- | --- | --- | --- | --- | --- | --- | --- | --- | --- | --- | --- | --- |
| **Definitions of time points and intervals** |  |  |  | | |  | |  | | |  | | |  | | | | |  | | | |  | | |  | | | |  | | | |  | |  | |  |
| For studies requiring the measurement of an interval, are the beginning and end points of this interval clearly defined? | Yes | Yes | Yes | | | Yes | | Yes | | | Yes | | | Yes | | | | | Yes | | | | Yes | | | Yes | | | | Yes | | | | Yes | | No | |  |
| For all time points and intervals described, are there precise, transparent and repeatable definitions, and is the complexity of time points such as the date of first symptom and date of first presentation addressed? | No | Yes | No | | | No | | Yes | | | No | | | Yes | | | | | Yes | | | | No | | | No | | | | No | | | | No | | No | |  |
| *For studies that require an estimate of the date of first symptom:* |  |  |  | | |  | |  | | |  | | |  | | | | |  | | | |  | | |  | | | |  | | | |  | |  | |  |
| Do the researchers refer to a theoretical framework underpinning definition of this time point? |  |  |  | | | No | |  | | |  | | |  | | | | |  | | | | No | | | No | | | | No | | | | No | | No | |  |
| Is there a discussion of the different biases influencing measurement of this time point? |  |  |  | | | No | |  | | |  | | |  | | | | |  | | | | Yes | | | No | | | | Yes | | | | No | | No | |  |
| *For studies that require measurement of a date of first presentation to healthcare:* |  |  |  | | |  | |  | | |  | | |  | | | | |  | | | |  | | |  | | | |  | | | |  | |  | |  |
| Do the researchers discuss the complexity of the date of first presentation? |  | Yes | No | | |  | |  | | |  | | |  | | | | | Yes | | | | No | | |  | | | | No | | | |  | | No | |  |
| *For studies that require measurement of a date of referral:* |  |  |  | | |  | |  | | |  | | |  | | | | |  | | | |  | | |  | | | |  | | | |  | |  | |  |
| Do the researchers discuss the nature of the referral and provide adequate detail - for example, whether it was for investigation or consultation by a colleague in secondary care? |  | Yes |  | | |  | | Yes | | |  | | |  | | | | |  | | | |  | | |  | | | | No | | | |  | |  | |  |
| *For studies that require measurement of the date of diagnosis:* |  |  |  | | |  | |  | | |  | | |  | | | | |  | | | |  | | |  | | | |  | | | |  | |  | |  |
| Do the researchers use an existing hierarchical rationale for the date of diagnosis measurement? | No | No | No | | | No | |  | | | No | | | No | | | | | No | | | | No | | | No | | | | No | | | | No | |  | |  |
| **Measurement** |  |  |  | | |  | |  | | |  | | |  | | | | |  | | | |  | | |  | | | |  | | | |  | |  | |  |
| Is the healthcare context in which the study is based fully described? | No | No | No | | | Yes | | Yes | | | No | | | Yes | | | | | Yes | | | | No | | | No | | | | No | | | | No | | No | |  |
| Do the questions on time points and/or intervals clearly derive from stated definitions? | No | Yes | No | | | No | | Yes | | | No | | | Yes | | | | | Yes | | | | Yes | | | No | | | | No | | | | No | | No | |  |
| Do researchers acknowledge the need for theoretical validation and make reference to the theoretical framework(s) underpinning measurement and analysis of the time points? | No | No | No | | | No | | No | | | No | | | Yes | | | | | Yes | | | | No | | | No | | | | No | | | | No | | No | |  |
| *For studies using questionnaires and/or interviews with patients and/or health-care providers:* |  |  |  | | |  | |  | | |  | | |  | | | | |  | | | |  | | |  | | | |  | | | |  | |  | |  |
| Has a validated instrument been used? |  |  |  | | | No | |  | | |  | | |  | | | | |  | | | |  | | |  | | | |  | | | |  | |  | |  |
| Have the researchers included a copy of their instrument? |  |  |  | | | No | |  | | |  | | |  | | | | |  | | | |  | | |  | | | |  | | | |  | |  | |  |
| Is there some discussion of how reliability and validity (trustworthiness) has been established? |  |  |  | | | No | |  | | |  | | |  | | | | |  | | | |  | | |  | | | |  | | | |  | |  | |  |
| Do researchers acknowledge the need for theoretical validation and make reference to the theoretical framework(s) underpinning measurement and analysis of the time points? |  |  |  | | | No | |  | | |  | | |  | | | | |  | | | |  | | |  | | | |  | | | |  | |  | |  |
| Is there discussion of the different biases influencing measurement of the time points, such as how and when the question is asked and who is being asked? |  |  |  | | | No | |  | | |  | | |  | | | | |  | | | |  | | |  | | | |  | | | |  | |  | |  |
| Is the timing of the interview in relation to the date of diagnosis provided? |  |  |  | | | Yes | |  | | |  | | |  | | | | |  | | | |  | | |  | | | |  | | | |  | |  | |  |
| Is there any triangulation of self-reported data with other data sources such as case notes? |  |  |  | | | No | |  | | |  | | |  | | | | |  | | | |  | | |  | | | |  | | | |  | |  | |  |
| Is data analysis described in full including how and why data are categorised, how missing and incomplete date are managed, and how outliers at both ends of the spectrum are accounted for? |  |  |  | | | No | |  | | |  | | |  | | | | |  | | | |  | | |  | | | |  | | | |  | |  | |  |
| *For studies using primary case-note audit and database analysis:* |  |  |  | | |  | |  | | |  | | |  | | | | |  | | | |  | | |  | | | |  | | | |  | |  | |  |
| Case-note analysis: is there a clear and precise description of how case-note data were used to ascertain time points with an acknowledgement of limitations of such data? | No | No | No | | |  | |  | | |  | | | Yes | | | | |  | | | | Yes | | | No | | | | No | | | | No | | No | |  |
| For database analysis: is there a thorough description of the database chosen including sampling coverage and completeness of information? | No | Yes |  | | |  | | Yes | | | Yes | | | Yes | | | | | Yes | | | |  | | |  | | | | No | | | |  | |  | |  |
| **Arhus Checklist cont…** | Redaniel 2014[46] | Gillis 2014[47] | de Sousa 2014[48] | | | Ben-Ishay 2013[49] | | Esteva 2013[50] | | | Taggarshe 2013[27] | | | | Kaplan 2013[51] | | | | | Deng 2012[52] | | | | Mukherji 2011[53] | | | Chan 2010[54] | | | Shabbir 2009[56] | | | | Tohme 2008[57] | | Johnston 2004[60] | |  |
| **Definitions of time points and intervals** |  |  |  | | |  | |  | | |  | | | |  | | | | |  | | | |  | | |  | | |  | | | |  | |  | |  |
| For studies requiring the measurement of an interval, are the beginning and end points of this interval clearly defined? | Yes | Yes | Yes | | | No | | Yes | | | No | | | | No | | | | | Yes | | | | No | | | Yes | | | No | | | | Yes | | Yes | |  |
| For all time points and intervals described, are there precise, transparent and repeatable definitions, and is the complexity of time points such as the date of first symptom and date of first presentation addressed? | Yes | Yes | No | | | No | | Yes | | | No | | | | No | | | | | No | | | | No | | | No | | | No | | | | No | | Yes | |  |
| *For studies that require an estimate of the date of first symptom:* |  |  |  | | |  | |  | | |  | | | |  | | | | |  | | | |  | | |  | | |  | | | |  | |  | |  |
| Do the researchers refer to a theoretical framework underpinning definition of this time point? |  |  | No | | | No | | Yes | | | No | | | | No | | | | | No | | | | No | | | No | | |  | | | | No | |  | |  |
| Is there a discussion of the different biases influencing measurement of this time point? |  |  | No | | | No | | Yes | | | No | | | | No | | | | | No | | | | No | | | No | | |  | | | | No | |  | |  |
| *For studies that require measurement of a date of first presentation to healthcare:* |  |  |  | | |  | |  | | |  | | | |  | | | | |  | | | |  | | |  | | |  | | | |  | |  | |  |
| Do the researchers discuss the complexity of the date of first presentation? |  |  |  | | |  | |  | | |  | | | |  | | | | |  | | | | No | | | No | | |  | | | |  | |  | |  |
| *For studies that require measurement of a date of referral:* |  |  |  | | |  | |  | | |  | | | |  | | | | |  | | | |  | | |  | | |  | | | |  | |  | |  |
| Do the researchers discuss the nature of the referral and provide adequate detail - for example, whether it was for investigation or consultation by a colleague in secondary care? |  |  |  | | |  | |  | | |  | | | |  | | | | |  | | | |  | | |  | | | Yes | | | | No | |  | |  |
| *For studies that require measurement of the date of diagnosis:* |  |  |  | | |  | |  | | |  | | | |  | | | | |  | | | |  | | |  | | |  | | | |  | |  | |  |
| Do the researchers use an existing hierarchical rationale for the date of diagnosis measurement? | Yes | No | No | | | No | | No | | | No | | | | No | | | | | No | | | |  | | |  | | |  | | | | No | | No | |  |
| **Measurement** |  |  |  | | |  | |  | | |  | | | |  | | | | |  | | | |  | | |  | | |  | | | |  | |  | |  |
| Is the healthcare context in which the study is based fully described? | No | Yes | No | | | No | | No | | | Yes | | | | No | | | | | Yes | | | | No | | | No | | | Yes | | | | No | | Yes | |  |
| Do the questions on time points and/or intervals clearly derive from stated definitions? | No | No | No | | | No | | Yes | | | No | | | | No | | | | | No | | | | No | | | No | | | Yes | | | | No | | Yes | |  |
| Do researchers acknowledge the need for theoretical validation and make reference to the theoretical framework(s) underpinning measurement and analysis of the time points? | No | No | No | | | No | | Yes | | | No | | | | No | | | | | No | | | | No | | | No | | | No | | | | No | | Yes | |  |
| *For studies using questionnaires and/or interviews with patients and/or health-care providers:* |  |  |  | | |  | |  | | |  | | | |  | | | | |  | | | |  | | |  | | |  | | | |  | |  | |  |
| Has a validated instrument been used? |  |  |  | | |  | | No | | |  | | | |  | | | | | No | | | |  | | |  | | |  | | | |  | |  | |  |
| Have the researchers included a copy of their instrument? |  |  |  | | |  | | Yes | | |  | | | |  | | | | | No | | | |  | | |  | | |  | | | |  | |  | |  |
| Is there some discussion of how reliability and validity (trustworthiness) has been established? |  |  |  | | |  | | Yes | | |  | | | |  | | | | | No | | | |  | | |  | | |  | | | |  | |  | |  |
| Do researchers acknowledge the need for theoretical validation and make reference to the theoretical framework(s) underpinning measurement and analysis of the time points? |  |  |  | | |  | | Yes | | |  | | | |  | | | | | No | | | |  | | |  | | |  | | | |  | |  | |  |
| Is there discussion of the different biases influencing measurement of the time points, such as how and when the question is asked and who is being asked? |  |  |  | | |  | | Yes | | |  | | | |  | | | | | Yes | | | |  | | |  | | |  | | | |  | |  | |  |
| Is the timing of the interview in relation to the date of diagnosis provided? |  |  |  | | |  | | Yes | | |  | | | |  | | | | | Yes | | | |  | | |  | | |  | | | |  | |  | |  |
| Is there any triangulation of self-reported data with other data sources such as case notes? |  |  |  | | |  | | Yes | | |  | | | |  | | | | | Yes | | | |  | | |  | | |  | | | |  | |  | |  |
| Is data analysis described in full including how and why data are categorised, how missing and incomplete date are managed, and how outliers at both ends of the spectrum are accounted for? |  |  |  | | |  | | Yes | | |  | | | |  | | | | | Yes | | | |  | | |  | | |  | | | |  | |  | |  |
| *For studies using primary case-note audit and database analysis:* |  |  |  | | |  | |  | | |  | | | |  | | | | |  | | | |  | | |  | | |  | | | |  | |  | |  |
| Case-note analysis: is there a clear and precise description of how case-note data were used to ascertain time points with an acknowledgement of limitations of such data? |  |  | No | | | No | | Yes | | | No | | | | No | | | | | No | | | | No | | | No | | | No | | | | No | | Yes | |  |
| For database analysis: is there a thorough description of the database chosen including sampling coverage and completeness of information? | Yes | Yes |  | | |  | |  | | | Yes | | | |  | | | | |  | | | |  | | |  | | |  | | | |  | | Yes | |  |
| **Aarhus Checklist cont…** | Robertson 2004[61] | Pocard 1997[63] | Heys 1994[64] | | Marble 1992[65] | | Pearson 2019[30] | | | Wanis 2017[29] | | Jones 2017[41] | | | | | Pita-Fernandez 2016[43] | | | | | Zhang 2015[44] | | | Porter 2005[58] | | | | Neal 2005[59] | | | Fadlouallah 2010[55] | | | | Sahraoui 2000[62] | | Lima 2021[81] |
| **Definitions of time points and intervals** |  |  |  | |  | |  | | |  | |  | | | | |  | | | | |  | | |  | | | |  | | |  | | | |  | |  |
| For studies requiring the measurement of an interval, are the beginning and end points of this interval clearly defined? | Yes | Yes | Yes | | Yes | | Yes | | | Yes | | Yes | | | | | Yes | | | | | Yes | | | Yes | | | | No | | | Yes | | | | Yes | | Yes |
| For all time points and intervals described, are there precise, transparent and repeatable definitions, and is the complexity of time points such as the date of first symptom and date of first presentation addressed? | Yes | No | No | | No | | Yes | | | Yes | | Yes | | | | | Yes | | | | | Yes | | | Yes | | | | No | | | No | | | | No | | No |
| *For studies that require an estimate of the date of first symptom:* |  |  |  | |  | |  | | |  | |  | | | | |  | | | | |  | | |  | | | |  | | |  | | | |  | |  |
| Do the researchers refer to a theoretical framework underpinning definition of this time point? |  | No | No | | No | |  | | |  | |  | | | | | No | | | | | Yes | | | No | | | | Yes | | | No | | | | No | |  |
| Is there a discussion of the different biases influencing measurement of this time point? |  | No | No | | No | |  | | |  | |  | | | | | Yes | | | | | Yes | | | Yes | | | | Yes | | | No | | | | No | |  |
| *For studies that require measurement of a date of first presentation to healthcare:* |  |  |  | |  | |  | | |  | |  | | | | |  | | | | |  | | |  | | | |  | | |  | | | |  | |  |
| Do the researchers discuss the complexity of the date of first presentation? | Yes |  |  | | No | |  | | |  | | Yes | | | | |  | | | | | Yes | | | Yes | | | |  | | |  | | | |  | |  |
| *For studies that require measurement of a date of referral:* |  |  |  | |  | |  | | |  | |  | | | | |  | | | | |  | | |  | | | |  | | |  | | | |  | |  |
| Do the researchers discuss the nature of the referral and provide adequate detail - for example, whether it was for investigation or consultation by a colleague in secondary care? |  |  |  | |  | | Yes | | |  | |  | | | | |  | | | | |  | | |  | | | | No | | |  | | | |  | |  |
| *For studies that require measurement of the date of diagnosis:* |  |  |  | |  | |  | | |  | |  | | | | |  | | | | |  | | |  | | | |  | | |  | | | |  | |  |
| Do the researchers use an existing hierarchical rationale for the date of diagnosis measurement? |  | No | No | | No | | Yes | | | No | |  | | | | | No | | | | |  | | | Yes | | | | No | | | No | | | |  | | No |
| **Measurement** |  |  |  | |  | |  | | |  | |  | | | | |  | | | | |  | | |  | | | |  | | |  | | | |  | |  |
| Is the healthcare context in which the study is based fully described? | Yes | No | No | | No | | Yes | | | Yes | | Yes | | | | | No | | | | | No | | | No | | | | No | | | No | | | | No | | Yes |
| Do the questions on time points and/or intervals clearly derive from stated definitions? | No | No | No | | No | | Yes | | | No | | No | | | | | No | | | | | No | | | No | | | | Yes | | | No | | | | No | | Yes |
| Do researchers acknowledge the need for theoretical validation and make reference to the theoretical framework(s) underpinning measurement and analysis of the time points? | No | No | No | | No | | Yes | | | No | | No | | | | | No | | | | | Yes | | | No | | | | Yes | | | No | | | | No | | No |
| *For studies using questionnaires and/or interviews with patients and/or health-care providers:* |  |  |  | |  | |  | | |  | |  | | | | |  | | | | |  | | |  | | | |  | | |  | | | |  | |  |
| Has a validated instrument been used? |  |  |  | |  | |  | | |  | | No | | | | |  | | | | | No | | | No | | | | Yes | | |  | | | |  | |  |
| Have the researchers included a copy of their instrument? |  |  |  | |  | |  | | |  | | Yes | | | | |  | | | | | No | | | Yes | | | | No | | |  | | | |  | |  |
| Is there some discussion of how reliability and validity (trustworthiness) has been established? |  |  |  | |  | |  | | |  | | No | | | | |  | | | | | No | | | Yes | | | | No | | |  | | | |  | |  |
| Do researchers acknowledge the need for theoretical validation and make reference to the theoretical framework(s) underpinning measurement and analysis of the time points? |  |  |  | |  | |  | | |  | | No | | | | |  | | | | | Yes | | | No | | | | Yes | | |  | | | |  | |  |
| Is there discussion of the different biases influencing measurement of the time points, such as how and when the question is asked and who is being asked? |  |  |  | |  | |  | | |  | | No | | | | |  | | | | | Yes | | | Yes | | | | Yes | | |  | | | |  | |  |
| Is the timing of the interview in relation to the date of diagnosis provided? |  |  |  | |  | |  | | |  | | Yes | | | | |  | | | | | Yes | | | Yes | | | | No | | |  | | | |  | |  |
| Is there any triangulation of self-reported data with other data sources such as case notes? |  |  |  | |  | |  | | |  | | Yes | | | | |  | | | | | Yes | | | Yes | | | | No | | |  | | | |  | |  |
| Is data analysis described in full including how and why data are categorised, how missing and incomplete date are managed, and how outliers at both ends of the spectrum are accounted for? |  |  |  | |  | |  | | |  | | No | | | | |  | | | | | No | | | No | | | | No | | |  | | | |  | |  |
| *For studies using primary case-note audit and database analysis:* |  |  |  | |  | |  | | |  | |  | | | | |  | | | | |  | | |  | | | |  | | |  | | | |  | |  |
| Case-note analysis: is there a clear and precise description of how case-note data were used to ascertain time points with an acknowledgement of limitations of such data? | Yes | No | No | | No | |  | | | Yes | | No | | | | | Yes | | | | | No | | | Yes | | | |  | | | No | | | | No | |  |
| For database analysis: is there a thorough description of the database chosen including sampling coverage and completeness of information? | Yes |  |  | |  | | Yes | | |  | |  | | | | |  | | | | |  | | |  | | | |  | | |  | | | |  | | Yes |
| **Aarhus Checklist cont…** | Da Silva 2020[67] | Rogers 2017[66] | Galadima 2021[71] | | | Delisle 2020[68] | | Di Leo 2020[69] | | | Webber 2020[74] | | Van Erp 2019[73] | | | | | Eaglehouse 2020[70] | | | | | | Rittitit 2020[72] | | | | Price 2020[75] | | | de Castro 2019[76] | | Bergin 2019[77] | |  | |  |  |
| **Definitions of time points and intervals** |  |  |  | | |  | |  | | |  | |  | | | | |  | | | | | |  | | | |  | | |  | |  | |  | |  |  |
| For studies requiring the measurement of an interval, are the beginning and end points of this interval clearly defined? | Yes | Yes | Yes | | | Yes | | Yes | | | Yes | | Yes | | | | | Yes | | | | | | Yes | | | | Yes | | | Yes | | Yes | |  | |  |  |
| For all time points and intervals described, are there precise, transparent and repeatable definitions, and is the complexity of time points such as the date of first symptom and date of first presentation addressed? | No | No | No | | | Yes | | No | | | Yes | | Yes | | | | | Yes | | | | | | Yes | | | | Yes | | | No | | Yes | |  | |  |  |
| *For studies that require an estimate of the date of first symptom:* |  |  |  | | |  | |  | | |  | |  | | | | |  | | | | | |  | | | |  | | |  | |  | |  | |  |  |
| Do the researchers refer to a theoretical framework underpinning definition of this time point? | No | No |  | | |  | | No | | |  | | Yes | | | | |  | | | | | | No | | | |  | | |  | | Yes | |  | |  |  |
| Is there a discussion of the different biases influencing measurement of this time point? | No | Yes |  | | |  | | Yes | | |  | | Yes | | | | |  | | | | | | Yes | | | |  | | |  | | Yes | |  | |  |  |
| *For studies that require measurement of a date of first presentation to healthcare:* |  |  |  | | |  | |  | | |  | |  | | | | |  | | | | | |  | | | |  | | |  | |  | |  | |  |  |
| Do the researchers discuss the complexity of the date of first presentation? |  |  |  | | | Yes | |  | | | Yes | | Yes | | | | |  | | | | | | No | | | | Yes | | | No | | Yes | |  | |  |  |
| *For studies that require measurement of a date of referral:* |  |  |  | | |  | |  | | |  | |  | | | | |  | | | | | |  | | | |  | | |  | |  | |  | |  |  |
| Do the researchers discuss the nature of the referral and provide adequate detail - for example, whether it was for investigation or consultation by a colleague in secondary care? |  |  |  | | |  | |  | | |  | | Yes | | | | |  | | | | | |  | | | |  | | |  | | No | |  | |  |  |
| *For studies that require measurement of the date of diagnosis:* |  |  |  | | |  | |  | | |  | |  | | | | |  | | | | | |  | | | |  | | |  | |  | |  | |  |  |
| Do the researchers use an existing hierarchical rationale for the date of diagnosis measurement? | No | No | No | | |  | | No | | | No | |  | | | | | Yes | | | | | | No | | | | No | | | No | | Yes | |  | |  |  |
| **Measurement** |  |  |  | | |  | |  | | |  | |  | | | | |  | | | | | |  | | | |  | | |  | |  | |  | |  |  |
| Is the healthcare context in which the study is based fully described? | No | No | Yes | | | Yes | | No | | | Yes | | Yes | | | | | Yes | | | | | | No | | | | Yes | | | Yes | | Yes | |  | |  |  |
| Do the questions on time points and/or intervals clearly derive from stated definitions? | No | No | No | | | Yes | | No | | | Yes | | Yes | | | | | Yes | | | | | | Yes | | | | Yes | | | Yes | | Yes | |  | |  |  |
| Do researchers acknowledge the need for theoretical validation and make reference to the theoretical framework(s) underpinning measurement and analysis of the time points? | No | No | No | | | No | | No | | | Yes | | Yes | | | | | No | | | | | | No | | | | Yes | | | No | | Yes | |  | |  |  |
| *For studies using questionnaires and/or interviews with patients and/or health-care providers:* |  |  |  | | |  | |  | | |  | |  | | | | |  | | | | | |  | | | |  | | |  | |  | |  | |  |  |
| Has a validated instrument been used? |  | Yes |  | | |  | |  | | |  | |  | | | | |  | | | | | | No | | | |  | | |  | | Yes | |  | |  |  |
| Have the researchers included a copy of their instrument? |  | No |  | | |  | |  | | |  | |  | | | | |  | | | | | | No | | | |  | | |  | | No | |  | |  |  |
| Is there some discussion of how reliability and validity (trustworthiness) has been established? |  | Yes |  | | |  | |  | | |  | |  | | | | |  | | | | | | No | | | |  | | |  | | Yes | |  | |  |  |
| Do researchers acknowledge the need for theoretical validation and make reference to the theoretical framework(s) underpinning measurement and analysis of the time points? |  | No |  | | |  | |  | | |  | |  | | | | |  | | | | | | No | | | |  | | |  | | Yes | |  | |  |  |
| Is there discussion of the different biases influencing measurement of the time points, such as how and when the question is asked and who is being asked? |  | Yes |  | | |  | |  | | |  | |  | | | | |  | | | | | | Yes | | | |  | | |  | | Yes | |  | |  |  |
| Is the timing of the interview in relation to the date of diagnosis provided? |  | Yes |  | | |  | |  | | |  | |  | | | | |  | | | | | | No | | | |  | | |  | | No | |  | |  |  |
| Is there any triangulation of self-reported data with other data sources such as case notes? |  | Yes |  | | |  | |  | | |  | |  | | | | |  | | | | | | Yes | | | |  | | |  | | Yes | |  | |  |  |
| Is data analysis described in full including how and why data are categorised, how missing and incomplete date are managed, and how outliers at both ends of the spectrum are accounted for? |  | No |  | | |  | |  | | |  | |  | | | | |  | | | | | | Yes | | | |  | | |  | | Yes | |  | |  |  |
| *For studies using primary case-note audit and database analysis:* |  |  |  | | |  | |  | | |  | |  | | | | |  | | | | | |  | | | |  | | |  | |  | |  | |  |  |
| Case-note analysis: is there a clear and precise description of how case-note data were used to ascertain time points with an acknowledgement of limitations of such data? | No | No | No | | |  | | No | | |  | | Yes | | | | |  | | | | | | No | | | |  | | | No | | Yes | |  | |  |  |
| For database analysis: is there a thorough description of the database chosen including sampling coverage and completeness of information? |  |  | Yes | | | Yes | |  | | | Yes | | Yes | | | | | Yes | | | | | |  | | | | Yes | | |  | | No | |  | |  |  |
|  |  |  |  | | |  | |  | | |  | |  | | | | |  | | | | | |  | | | |  | | |  | |  | |  | |  |  |
| **Aarhus Checklist cont…** | Majano 2021[79] | | | Foppa 2021[78] | | | | | Johnson 2021[80] | | | | | | |  | | | | |  |  |  |  |  |  |  |  |  |  |  |  |  |  |  |  |  |  |
| **Definitions of time points and intervals** |  | | |  | | | | |  | | | | | | |  | | | | |  |  |  |  |  |  |  |  |  |  |  |  |  |  |  |  |  |  |
| For studies requiring the measurement of an interval, are the beginning and end points of this interval clearly defined? | Yes | | | Yes | | | | | Yes | | | | | | |  | | | | |  |  |  |  |  |  |  |  |  |  |  |  |  |  |  |  |  |  |
| For all time points and intervals described, are there precise, transparent and repeatable definitions, and is the complexity of time points such as the date of first symptom and date of first presentation addressed? | Yes | | | No | | | | | Yes | | | | | | |  | | | | |  |  |  |  |  |  |  |  |  |  |  |  |  |  |  |  |  |  |
| *For studies that require an estimate of the date of first symptom:* |  | | |  | | | | |  | | | | | | |  | | | | |  |  |  |  |  |  |  |  |  |  |  |  |  |  |  |  |  |  |
| Do the researchers refer to a theoretical framework underpinning definition of this time point? | No | | | No | | | | |  | | | | | | |  | | | | |  |  |  |  |  |  |  |  |  |  |  |  |  |  |  |  |  |  |
| Is there a discussion of the different biases influencing measurement of this time point? | Yes | | | No | | | | |  | | | | | | |  | | | | |  |  |  |  |  |  |  |  |  |  |  |  |  |  |  |  |  |  |
| *For studies that require measurement of a date of first presentation to healthcare:* |  | | |  | | | | |  | | | | | | |  | | | | |  |  |  |  |  |  |  |  |  |  |  |  |  |  |  |  |  |  |
| Do the researchers discuss the complexity of the date of first presentation? |  | | |  | | | | |  | | | | | | |  | | | | |  |  |  |  |  |  |  |  |  |  |  |  |  |  |  |  |  |  |
| *For studies that require measurement of a date of referral:* |  | | |  | | | | |  | | | | | | |  | | | | |  |  |  |  |  |  |  |  |  |  |  |  |  |  |  |  |  |  |
| Do the researchers discuss the nature of the referral and provide adequate detail - for example, whether it was for investigation or consultation by a colleague in secondary care? |  | | |  | | | | |  | | | | | | |  | | | | |  |  |  |  |  |  |  |  |  |  |  |  |  |  |  |  |  |  |
| *For studies that require measurement of the date of diagnosis:* |  | | |  | | | | |  | | | | | | |  | | | | |  |  |  |  |  |  |  |  |  |  |  |  |  |  |  |  |  |  |
| Do the researchers use an existing hierarchical rationale for the date of diagnosis measurement? | Yes | | | No | | | | | No | | | | | | |  | | | | |  |  |  |  |  |  |  |  |  |  |  |  |  |  |  |  |  |  |
| **Measurement** |  | | |  | | | | |  | | | | | | |  | | | | |  |  |  |  |  |  |  |  |  |  |  |  |  |  |  |  |  |  |
| Is the healthcare context in which the study is based fully described? | Yes | | | No | | | | | Yes | | | | | | |  | | | | |  |  |  |  |  |  |  |  |  |  |  |  |  |  |  |  |  |  |
| Do the questions on time points and/or intervals clearly derive from stated definitions? | Yes | | | No | | | | | No | | | | | | |  | | | | |  |  |  |  |  |  |  |  |  |  |  |  |  |  |  |  |  |  |
| Do researchers acknowledge the need for theoretical validation and make reference to the theoretical framework(s) underpinning measurement and analysis of the time points? | No | | | No | | | | | No | | | | | | |  | | | | |  |  |  |  |  |  |  |  |  |  |  |  |  |  |  |  |  |  |
| *For studies using questionnaires and/or interviews with patients and/or health-care providers:* |  | | |  | | | | |  | | | | | | |  | | | | |  |  |  |  |  |  |  |  |  |  |  |  |  |  |  |  |  |  |
| Has a validated instrument been used? |  | | |  | | | | |  | | | | | | |  | | | | |  |  |  |  |  |  |  |  |  |  |  |  |  |  |  |  |  |  |
| Have the researchers included a copy of their instrument? |  | | |  | | | | |  | | | | | | |  | | | | |  |  |  |  |  |  |  |  |  |  |  |  |  |  |  |  |  |  |
| Is there some discussion of how reliability and validity (trustworthiness) has been established? |  | | |  | | | | |  | | | | | | |  | | | | |  |  |  |  |  |  |  |  |  |  |  |  |  |  |  |  |  |  |
| Do researchers acknowledge the need for theoretical validation and make reference to the theoretical framework(s) underpinning measurement and analysis of the time points? |  | | |  | | | | |  | | | | | | |  | | | | |  |  |  |  |  |  |  |  |  |  |  |  |  |  |  |  |  |  |
| Is there discussion of the different biases influencing measurement of the time points, such as how and when the question is asked and who is being asked? |  | | |  | | | | |  | | | | | | |  | | | | |  |  |  |  |  |  |  |  |  |  |  |  |  |  |  |  |  |  |
| Is the timing of the interview in relation to the date of diagnosis provided? |  | | |  | | | | |  | | | | | | |  | | | | |  |  |  |  |  |  |  |  |  |  |  |  |  |  |  |  |  |  |
| Is there any triangulation of self-reported data with other data sources such as case notes? |  | | |  | | | | |  | | | | | | |  | | | | |  |  |  |  |  |  |  |  |  |  |  |  |  |  |  |  |  |  |
| Is data analysis described in full including how and why data are categorised, how missing and incomplete date are managed, and how outliers at both ends of the spectrum are accounted for? |  | | |  | | | | |  | | | | | | |  | | | | |  |  |  |  |  |  |  |  |  |  |  |  |  |  |  |  |  |  |
| *For studies using primary case-note audit and database analysis:* |  | | |  | | | | |  | | | | | | |  | | | | |  |  |  |  |  |  |  |  |  |  |  |  |  |  |  |  |  |  |
| Case-note analysis: is there a clear and precise description of how case-note data were used to ascertain time points with an acknowledgement of limitations of such data? |  | | | No | | | | | No | | | | | | |  | | | | |  |  |  |  |  |  |  |  |  |  |  |  |  |  |  |  |  |  |
| For database analysis: is there a thorough description of the database chosen including sampling coverage and completeness of information? | Yes | | |  | | | | |  | | | | | | |  | | | | |  |  |  |  |  |  |  |  |  |  |  |  |  |  |  |  |  |  |
